# Assessment of the Deepwater Horizon oil spill impact on Gulf coast microbial communities

**DOI:** 10.3389/fmicb.2014.00130

**Published:** 2014-04-03

**Authors:** Regina Lamendella, Steven Strutt, Sharon Borglin, Romy Chakraborty, Neslihan Tas, Olivia U. Mason, Jenni Hultman, Emmanuel Prestat, Terry C. Hazen, Janet K. Jansson

**Affiliations:** ^1^Lawrence Berkeley National Laboratory, Earth Sciences Division, Ecology DepartmentBerkeley, CA, USA; ^2^Biology Department, Juniata CollegeHuntingdon, PA, USA; ^3^Department of Earth, Ocean and Atmospheric Science, Florida State UniversityTallahassee, FL, USA; ^4^Department of Food Hygiene and Environmental Health, University of HelsinkiHelsinki, Finland; ^5^Department of Civil and Environmental Engineering, University of TennesseeKnoxville, TN, USA; ^6^Oak Ridge National Laboratory, Biosciences DivisionOak Ridge, TN, USA; ^7^Department of Energy, Joint Genome InstituteWalnut Creek, CA, USA

**Keywords:** hydrocarbons, 16S rRNA gene, metatranscriptomics, oil spill, microbial communities

## Abstract

One of the major environmental concerns of the Deepwater Horizon oil spill in the Gulf of Mexico was the ecological impact of the oil that reached shorelines of the Gulf Coast. Here we investigated the impact of the oil on the microbial composition in beach samples collected in June 2010 along a heavily impacted shoreline near Grand Isle, Louisiana. Successional changes in the microbial community structure due to the oil contamination were determined by deep sequencing of 16S rRNA genes. Metatranscriptomics was used to determine expression of functional genes involved in hydrocarbon degradation processes. In addition, potential hydrocarbon-degrading Bacteria were obtained in culture. The 16S data revealed that highly contaminated samples had higher abundances of *Alpha*- and *Gammaproteobacteria* sequences. Successional changes in these classes were observed over time, during which the oil was partially degraded. The metatranscriptome data revealed that PAH, n-alkane, and toluene degradation genes were expressed in the contaminated samples, with high homology to genes from *Alteromonadales*, *Rhodobacterales*, and *Pseudomonales*. Notably, *Marinobacter* (*Gammaproteobacteria*) had the highest representation of expressed genes in the samples. A *Marinobacter* isolated from this beach was shown to have potential for transformation of hydrocarbons in incubation experiments with oil obtained from the Mississippi Canyon Block 252 (MC252) well; collected during the Deepwater Horizon spill. The combined data revealed a response of the beach microbial community to oil contaminants, including prevalence of Bacteria endowed with the functional capacity to degrade oil.

## Introduction

The Deepwater Horizon oil spill was the largest accidental marine oil spill in the history of the oil industry, spewing an estimated 4.1 million barrels of crude oil into the Gulf of Mexico (Zukunft, 2010). In addition, 1.84 million gallons of chemical dispersants were applied to assist in oil dispersal (The Federal Intragency Solutions Group: Oil Budget Calculator Science and Engineering Team, 2010). Physical barriers, direct collection from the wellhead, skimming, and burning were also implemented in order to mitigate the effects of the spill. Despite significant efforts to protect hundreds of miles of beaches, wetlands, and estuaries from oil, it began washing up on the Gulf Coast by early May 2010 (OSAT-2, 2011). Most recent estimates indicate that up to 22% of the 4.1 million barrels of oil was either trapped under the surface of the water as sheen, carried on the water surface as conglomerated tar (Lubchenco et al., 2010; Kimes et al., 2013), or deposited onto surface sediments (US Coast Guard, USGS, and NOAA, 2010; Mason et al., 2004). Some of the oil washed ashore where it was either collected or became entrained in sand and sediments. The contamination of beach ecosystems raised considerable concern due to the potential for detrimental environmental and economic impacts in the Gulf region (McCrea-Strub et al., 2011; Sumaila et al., 2012).

Initial research studies of the Gulf oil spill mainly focused on the fate of the oil in the water column. These studies highlighted the significant contribution of microorganisms toward the degradation of oil in a deep-sea hydrocarbon plume (Camilli et al., 2010; Hazen et al., 2010; Valentine et al., 2010, 2012; Redmond and Valentine, 2011; Baelum et al., 2012; Mason et al., 2012), and in particular a rapid response of members of the *Gammaproteobacteria* to hydrocarbon inputs. Specifically, there was an initial increase in relative abundance of members of the *Oceanospirillales* (Hazen et al., 2010; Redmond and Valentine, 2011; Mason et al., 2012), followed by members of the genera *Colwellia* and *Cycloclasticus* during later sampling periods (Redmond and Valentine, 2011; Valentine et al., 2012; Dubinsky et al., 2013).

Comparably less is known about the fate of the oil that reached the shore during the Deepwater Horizon spill. One study by Kostka et al. (2011) investigated the impact of the oil on beach samples collected several months after the spill occurred (July and September 2010) at municipal Pensacola Beach, Florida. By 16S rRNA gene sequencing, the authors found that the spill had a significant impact on the abundance and community composition of indigenous bacteria in beach sand with increases in many members of the *Alpha*- and *Gammaproteobacteria*, including some well-known hydrocarbon degraders (*Alcanivorax* and *Marinobacter*) (Yakimov et al., 1998; Alonso-Gutiérrez et al., 2009). In the same study, several proteobacterial isolates, capable of growth on oil as their sole carbon source, were obtained from the contaminated samples (Kostka et al., 2011).

Here we aimed to determine the response of indigenous beach microbial communities to the oil that washed ashore early in the spill history. We focused our efforts on Elmers's Beach, Grand Isle, LA. This location was one of the most heavily oiled beaches in the Gulf, where oil began washing up onto the beach in early May 2010 (OSAT-2, 2011). A total of 153 oil contaminated and uncontaminated samples were collected at three time points in June 2010, while the oil continued to accumulate on the beach. The well was finally capped on July 15, 2010 and declared sealed on September 19, 2010.

We performed targeted 16S rRNA gene sequencing and total RNA sequencing (metatranscriptomics) to determine the composition of the microbial community, as well as to elucidate which members were actively degrading hydrocarbons in oiled samples. In addition, we isolated putative MC252 oil degrading microorganisms and studied their potential for hydrocarbon degradation. This study revealed a succession in the microbial community structure on the beach during early time points in the Deepwater Horizon oil spill. This study also represents the first use of metatranscriptome data to highlight the expression of genes involved in hydrocarbon transformations in a coastline community.

## Materials and methods

### Sample collection

Beach sand cores were collected on Elmer's Beach (29.1782853, −90.0684072) at three time points on 03/06/2010 (*n* = 7), 21/06/2010 (*n* = 7), and 29/06/2010 (*n* = 3). Sand cores (10–20 cm deep) were taken by manual insertion of 40 cm long polybutyrate plastic liners into the sand. The cores were taken from locations submerged in the water close to the waterline, at the waterline, and inland. To circumvent potential contamination from the polybutyrate liners, each sand core was sub-cored using a 25 mm diameter sterile copper pipe, and sectioned into 3 cm depth intervals. Additionally, tar-like samples found on the surface of the beach (*n* = 24) were collected at each sampling period by aseptically scraping approximately 2–10 g into sterile 50 mL conical tubes. All samples were kept on ice in the field and were maintained at 4°C until further processing. Detailed information about all the samples, relating to location, date, core depth, and hydrocarbon composition can be found in Supplemental Table S1.

### Acridine orange direct counts

Approximately 1 g of each sample was homogenized and diluted in 1X PBS. Samples were filtered through a 0.2 μm pore size black polycarbonate membrane (Whatman International Ltd., Piscataway, NJ). Filtered cells were stained with 25 mg/mL acridine orange for 2 min in the dark. Unbound acridine orange was filtered through the membrane with 10 mL filter sterilized 1X PBS (Sigma Aldrich Corp., St. Louis, MI) and the rinsed membrane was mounted on a slide for microscopy. Cells were imaged with a FITC filter on a Zeiss Axioskop (Carl Zeiss, Inc., Germany).

### Petroleum hydrocarbon concentrations

Total petroleum hydrocarbon (TPH) concentrations were determined using previously published procedures (Hazen et al., 2010) with the following modifications: 500 μL of chloroform were added to 500 mg of sample and then vortexed thoroughly, shaken for 2 min and sonicated for 2 min. The samples were incubated at room temperature for 1 h, centrifuged at 2,000 rpm for 5 min, and 50 μL of the extract was removed for analysis on an Agilent 6890N GC/FID (Santa Clara, CA). The GC was operated with an injector temperature of 250°C and detector temperature of 300°C, following a temperature program of 50°C for 2 min, ramped by 5°C/min until reaching 300°C and subsequently held for 15 min. TPH were quantified by integrating all the peaks from 20 to 60 min and comparing to oil standards (0–200 mg/L) obtained from the Macondo source well during the Deepwater Horizon spill.

PAH and alkane compound analysis was completed on the Agilent 6890N equipped with a 5972 mass selective detector and operated in SIM/SCAN mode. The injection temperature was 250°C, detector temperature was 300°C, and column used was 60 m Agilent HP-1 MS with a flow rate of 2 mL/min. The oven temperature program included a 50°C hold for 3 min ramped to 300°C at 4°C/min with a final 10 min hold at 300°C. Compound identification was determined from selective ion monitoring coupled with comparison to known standards and compound spectra in the NIST 08 MS library. Biomarker profiles were obtained by running the same samples in SIM mode targeting ions 191 for hopanes and 217 for steranes. Monitoring these ions has been widely used for oil source identification and degree of biodegradation (Venosa et al., 1997; Volkman et al., 1983; Greenwood and Georges, 1999; Hauser et al., 1999; Rosenbauer et al., 2010) and was utilized here to compare oil biomarker fingerprint to oil from the MC 252 source oil (Macondo crude). A proxy for biodegradation within the samples was calculated using the depletion of C_25_ with respect to C_17_ and the ratio of branched to aliphatic alkanes.

### DNA extraction

Samples were extracted in duplicate using a modified Miller DNA extraction method (Miller et al., 1999). Approximately 0.5 g of each sample was placed into an FT500-ND Pulse Tube (Pressure BioSciences, Inc., USA). 300 μL of Miller phosphate buffer and 300 μL of Miller SDS lysis buffer were added and mixed. 600 μL phenol:chloroform:isoamyl alcohol (25:24:1) were then added, and the tubes were subjected to 25 cycles of 35,000 psi for 10 s and ambient pressure for 10 s, to improve cell lysis. The mixture was transferred to a Lysing Matrix E tube (MP Biomedicals, Solon, OH) and subjected to bead-beating at 5.5 m/s for 45 s in a FastPrep (MP Biomedicals, Solon, OH) instrument. The tubes were centrifuged at 16,000 × g for 5 min at 4°C and 540 μL of supernatant was transferred to a 2 mL tube with addition of an equal volume of chloroform. Tubes were mixed and then centrifuged at 10,000 × g for 5 min, after which 400 μL of the aqueous phase was transferred to another tube and 2 volumes of Solution S3 (MoBio, Carlsbad, CA) were added and mixed by inversion. The subsequent clean-up methods were based on the MoBio Soil DNA extraction kit according to the manufacturer's instructions. Samples were recovered in 60 μL 10 mM Tris and stored at −20°C.

### Community profiling and statistical methods

Small subunit (SSU) rRNA gene sequences were amplified from duplicate DNA extractions using the primer pair 926f/1392r as previously described (Kunin et al., 2010). The reverse primer included a 5 bp barcode for multiplexing of samples during sequencing. Emulsion PCR and sequencing of the PCR amplicons was performed at DOE's Joint Genome Institute following manufacturer's instructions for the Roche 454 GS FLX Titanium technology, with the exception that the final dilution was 1e-8 (Allgaier et al., 2010).

Sequence reads were submitted to the PyroTagger computational pipeline (Kunin and Hugenholtz, 2010) where the reads were quality filtered, trimmed, dereplicated and clustered at 97% sequence identity. OTU tables generated from Pyrotagger were then imported into the QIIME pipeline (Caporaso et al., 2010) for further analyses. The number of sequence reads in each sample varied, therefore, the dataset was rarified. Alpha diversity calculations were performed on rarified data.

Multivariate community analysis was performed within PCORD 5 software (McCune et al., 2002) using normalized OTU data (family-level and OTU level). OTUs found in less than two samples were removed. Outliers were removed from the dataset using PCORD 5 with a cutoff of two standard deviations. The Bray-Curtis distance measure was used for non-metric multidimensional scaling (nMDS). Pearson correlation coefficients were calculated for metadata variables and each axis of the nMDS.

### RNA extraction, amplification, and sequencing

Total RNA was extracted from three of the oil contaminated samples as previously described (Kasai et al., 2001) and amplified using the Message Amp II-Bacteria Kit (Ambion, Austin, TX) following the manufacturer's instructions. First strand synthesis of cDNA from the resulting antisense RNA was carried out with the SuperScript III First Strand Synthesis System (Invitrogen, Carlsbad, CA). The SuperScript Double-Stranded cDNA Synthesis Kit (Invitrogen, Carlsbad, CA) was used to synthesize double stranded cDNA. cDNA was purified using a QIAquick PCR purification kit (Qiagen, Valencia, CA) and poly(A) tails were removed by digesting purified DNA with *Bpm*I for 3 h at 37°C. Digested cDNA was purified with QIAquick PCR purification kit (Qiagen, Valencia, CA). RNA was prepared for sequencing using the Illumina Truseq kit following the manufacturer's guidelines. Each library was sequenced on one lane of the Illumina HiSeq platform using the 150 bp Paired-end technology resulting in a total of 57 Gb of sequence data for all three samples.

### Metatranscriptomics data analysis

Raw Illumina sequence reads from each of three surface-contaminated samples (one from each sampling date) were trimmed using the CLC Genomics Workbench v5.0.1 with a quality score limit of 0.05. Phred quality scores (Q) were imported into the genomics workbench, where they were converted to error probabilities, using p_error_ = 10^Q/−10^ and were trimmed using a limit of 0.05 as described in the CLC Workbench Manual (http://www.clcbio.com).

Sequences shorter than 50 bp in length and all adapter sequences were removed. To characterize the active microbial community members, unassembled reads were searched against the Greengenes (DeSantis et al., 2006) database of 16S rRNA genes using BLASTn with a bit score cutoff of >100.

Transcript profiles from each sample were determined by first subjecting trimmed unassembled reads from each sample to ORF calling using Prodigal (Hyatt et al., 2010). Resulting ORFs were compared to a translated in-house hydrocarbon gene database using BLASTp. This database was constructed using all KEGG genes involved in hydrocarbon degradation from the KEGG database (Kanehisa and Goto, 2000). For the resulting BLAST outputs, the highest bit score was selected (min bit score >40). Metatranscriptome data from each sample were normalized to RecA expression levels. A pairwise statistical comparison of the BLAST analyses was carried out using STAMP (Parks and Beiko, 2010) using a two-sided Chi-square test (with Yates correction) statistic with the DP: Asymptotic-CC confidence interval method and the Bonferroni multiple test correction. A *p*-value of < 0.05 was used with a double effect size filter (difference between proportions effect size < 1.00 and a ratio of proportions effect size < 2.00. The metatranscriptome from the June 29 sampling date yielded an insufficient number of transcripts after quality filtering, thus subsequent analyses of the metatranscriptome data focused on the June 3 and June 21 samples.

Paired-end Illumina reads from each of the June 3 and June 21 samples were assembled using the *De Novo* Assembly Tool within the CLC Genomics Workbench at a word size of 20 and a bubble size of 50. Reads were scaffolded onto the contigs, which were submitted to MG-RAST (Meyer et al., 2008) for annotation. In MG-RAST, functional tables were generated for each sample against the KO annotation database, using default parameters (1e-5 maximum *e*-value cutoff, 60% minimum sequence identity, and 15 bp of minimum alignment length). To determine which organisms express genes involved in hydrocarbon degradation, contigs for each enzyme mapping to a xenobiotic pathway were annotated against the M5NR database for best-hit organismal classification using the default parameters. Using default parameters for the best-hit classification tool in MG-RAST, contigs were annotated against the Greengenes database to further assess presence of microbial community members through 16S rRNA transcripts. Recruitment plots were generated using a maximum e-value cutoff of 1e-3 and a log_2_ abundance scale. Contigs mapping to xenobiotic pathways were rarefied to a depth of 20,000 annotated contigs each. Xenobiotic degradation maps annotated using Kegg Orthology (KO) were downloaded from the KEGG server in KGML format and manually colored using the KGML editor (Klukas and Schreiber, 2007). Charts were generated from the Krona template (Ondov et al., 2011).

Assembled data are publicly available in the MG-RAST database under project ID 7309. Raw reads were submitted to NCBI's sequence read archive under project ID SUB442498.

### Enrichments and isolations

Bacteria were isolated from sand cores and contaminated beach samples after incubation under aerobic conditions with 100 ppm Macondo oil (MC 252) in either Marine broth medium (Difco), Minimal marine medium (Baelum et al., 2012) or Synthetic minimal marine medium. Synthetic Minimal marine medium was prepared as follows: For 1 L, autoclaved separately 850 mL of 20 g NaCl, 0.67 g KCl, 10 mL each of mineral and vitamin mixes (Coates et al., 1995), 100 mL of 30 mM phosphate buffer (pH 7.5), and added to 50 mL of 10.1 MgCl_2_.6H_2_O + 1.52 g CaCl_2_. 2 H_2_O. Enrichments that resulted in an increase in turbidity, in addition to an increase in cell number by microscopic observations, were transferred periodically into fresh media. After 3–4 transfers, colonies were obtained by plating on the respective agar plates and were incubated for 1 week. Isolates were obtained from single colonies and incubated aerobically in modified Synthetic Seawater medium with 100 ppm MC252 oil as the sole carbon source. Within a few days, the oil initially observed as a thin layer floating on top disappeared with a concurrent increase in cell number. At this point, DNA was extracted from the cultures using the MoBio UltraClean Microbial DNA Isolation Kit (MoBio Inc, Carlsbad, CA). PCR amplification was conducted using universal bacterial 16S rRNA gene primers 27F and 1492R in 50 ul reactions, with a final concentration of 0.025 unit/μl Taq, 0.2 mM dNTPs, 15 ng of DNA template, and 0.04 μM primer. Initial denaturation was at 95°C for 180 s, followed by 25 cycles of melting at 95°C for 30 s, annealing at 54°C for 30 s, extension at 72°C for 60 s. This was followed by a final extension of 10 min at 72°C and samples were held at 4°C on completion of amplification. Verified 16S amplicons were purified using the procedure provided in the MoBio Ultraclean PCR Clean-up kit (MoBio, Carlsbad, CA). Samples were sequenced using the Applied Biosystems ABI 3730XL DNA Analyzers with the BigDye Terminator V3.1 Cycle Sequencing Kit (Applied Biosystems, Carlsbad, CA), according to the manufacturer's instructions.

### Oil degradation with isolates

Different selective minimal media were prepared to test individual isolates for their ability to degrade oil, since the isolates belonged to different genera and had different nutritional requirements. *Marinobacter* isolate 33 was grown in MC252 oil amended with minimal marine media. *Roseobacter* isolate 36 was grown in modified Sistrom's Minimal Medium (Sistrom, 1962). Oil degradation experiments were set up in 30 mL of respective media amended with 20 ppm MC252 oil and 0.1 ppm COREXIT 9500, inoculated with the respective bacterial cultures, and incubated at room temperature in the dark. The inoculant was grown in the respective minimal medium amended with 0.1% Yeast extract to promote biomass. Prior to inoculation, cells were pelleted and washed in phosphate buffer (pH 7.5) to remove any carry over of media constituents. Heat killed cells (autoclaved) were used as negative controls, by 10% inoculation into experimental bottles containing oil and media.

At periodic intervals during the incubations, experimental bottles were sacrificed for hydrocarbon analyses to determine the extent of oil degradation. All glassware used in extraction and analyses was muffled at 500°C for 4 h prior to use. To extract hydrocarbons, the entire culture volume (30 ml) was transferred from the experimental bottles to a 50 mL glass culture tube with a Teflon-lined lid. The empty bottles were extracted three times with 2 mL of chloroform (BDH, ACS grade) to assay hydrocarbons sorbed to the glass and the rinses were added to the 50 mL tube. This mixture was vortexed for 1 min and extracted for 1 h, after which they were re-vortexed and centrifuged at 2000 rpm for 15 min to aid the separation of the chloroform from the aqueous media layer. The chloroform layer was removed with a glass pipette into a GC vial and analyzed as described above.

## Results

### State of the sampling site

On June 3, 2010, the sampling site was almost completely covered to the tidal berm with viscous oil. Seawater washing up on the shore contained large, amorphous globules of oil. On June 21, 2010, the beach no longer contained visible globules of oil and the surface of the sampling site was no longer covered in oil. Instead, the oil present was in the form of small dried globules, less than 2 cm in diameter. By June 29, 2010, oil and oil mixed with foam were evident at the sampling site. The beach surface was rust in color and a light sheen of oil was noted on the seawater surface.

### Chemical analysis

The hydrocarbon profiles of the beached oil and contaminated sand core samples showed a clear correspondence to the MC252 oil (Supplemental Figure. S1). Total petroleum hydrocarbons (TPH) ranged from 0 mg/kg to 2072 mg/kg. Several components in the oil decreased over time and were significantly depleted by June 21 and June 29 sampling dates. Specifically, there was a depletion of shorter alkanes (C_17_–C_20_) and a corresponding higher relative amount of longer chain alkanes (>C_20_) and branched alkanes. Cluster analysis of hydrocarbons revealed a clustering of the samples according to the level of hydrocarbon contamination (Supplemental Figure. S2). PAHs were detected in more than one third of the contaminated samples. Three-ring PAHs including, fluorene, anthracene, and phenanthracene and four-ring PAHs, including chrysene and pyrene, were highest in concentration of the measured PAH compounds, while naphthalene and other two-ring compounds were present in lower amounts and were nearly completely depleted in the less contaminated and uncontaminated samples from all time points (Supplemental Figure S2).

### Microbial community analyses

Cell counts ranged from 10^5^ cells g^−1^ in uncontaminated samples to more than 10^7^ cells g^−1^ in highly contaminated, beached oil samples and this difference was significant (*t*-test; *p* = 2.97 × 10^−5^). Therefore, there was a significant increase in microbial cell density as a result of the hydrocarbon influx on the beach, as previously reported by (Kostka et al., 2011).

**Figure 1:**
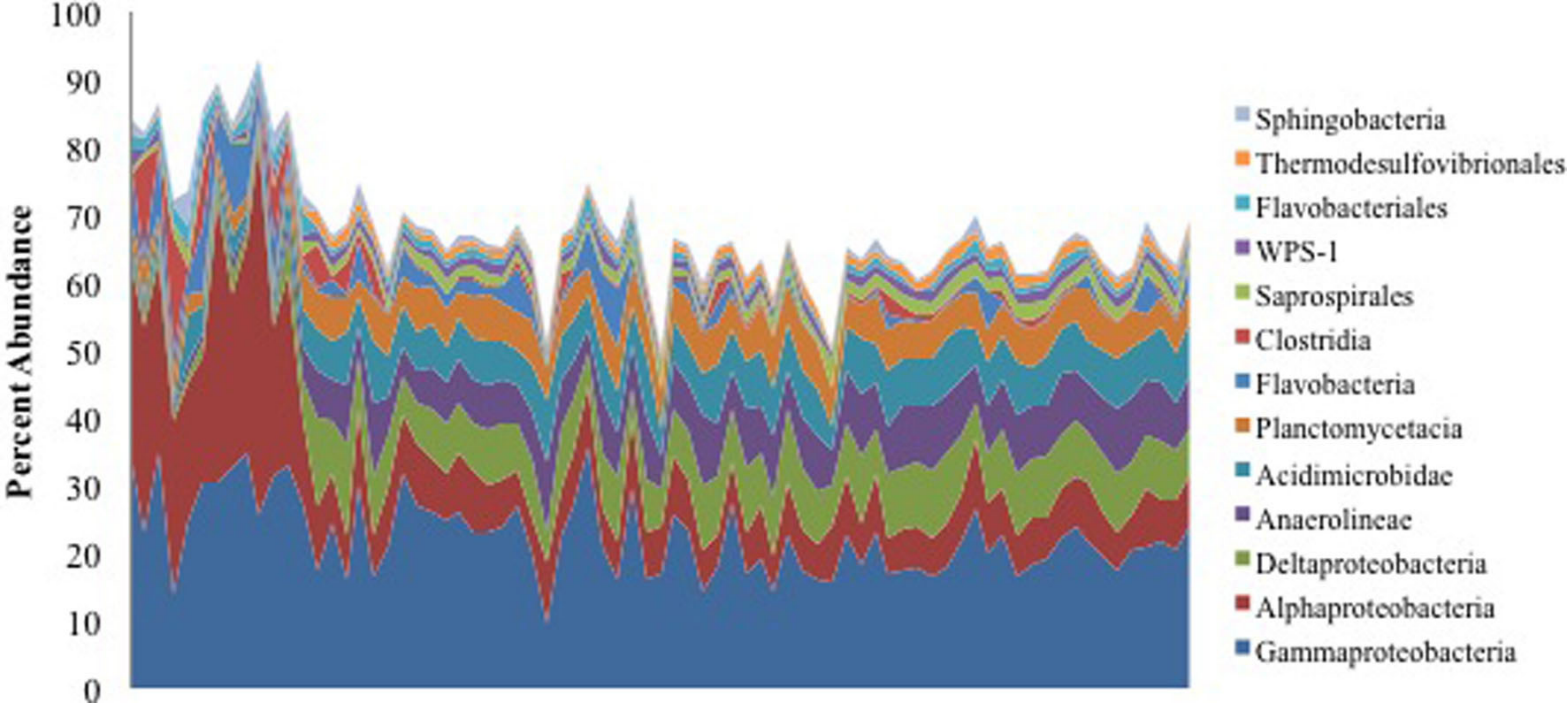
**Percent abundance of the 13 most abundant bacterial classes using 16S rRNA gene sequences**. Samples are ordered from highest to lowest TPH concentration, left to right.

We retrieved >1.6 million non-chimeric, quality filtered 16S rRNA gene sequences from a total of 153 oiled and uncontaminated samples, yielding more than 11,000 sequences per sample. The sequence data were dominated by OTUs corresponding to *Alpha*- and *Gammaproteobacteria* (Figure 1). Several OTUs that were abundant in the oil-contaminated samples corresponded to taxa with members known to degrade hydrocarbons, including *Rhodobacteraceae*, *Alteromondaceae*, *Pseudomonadaceae*, *Chromatiaceae, Alcanivoraceae*, and other families within the *Oceanospiralles*. Samples with the highest concentrations of hydrocarbons had higher relative abundances of *Alphaproteobacteria* (Figure 1).

**Figure 2:**
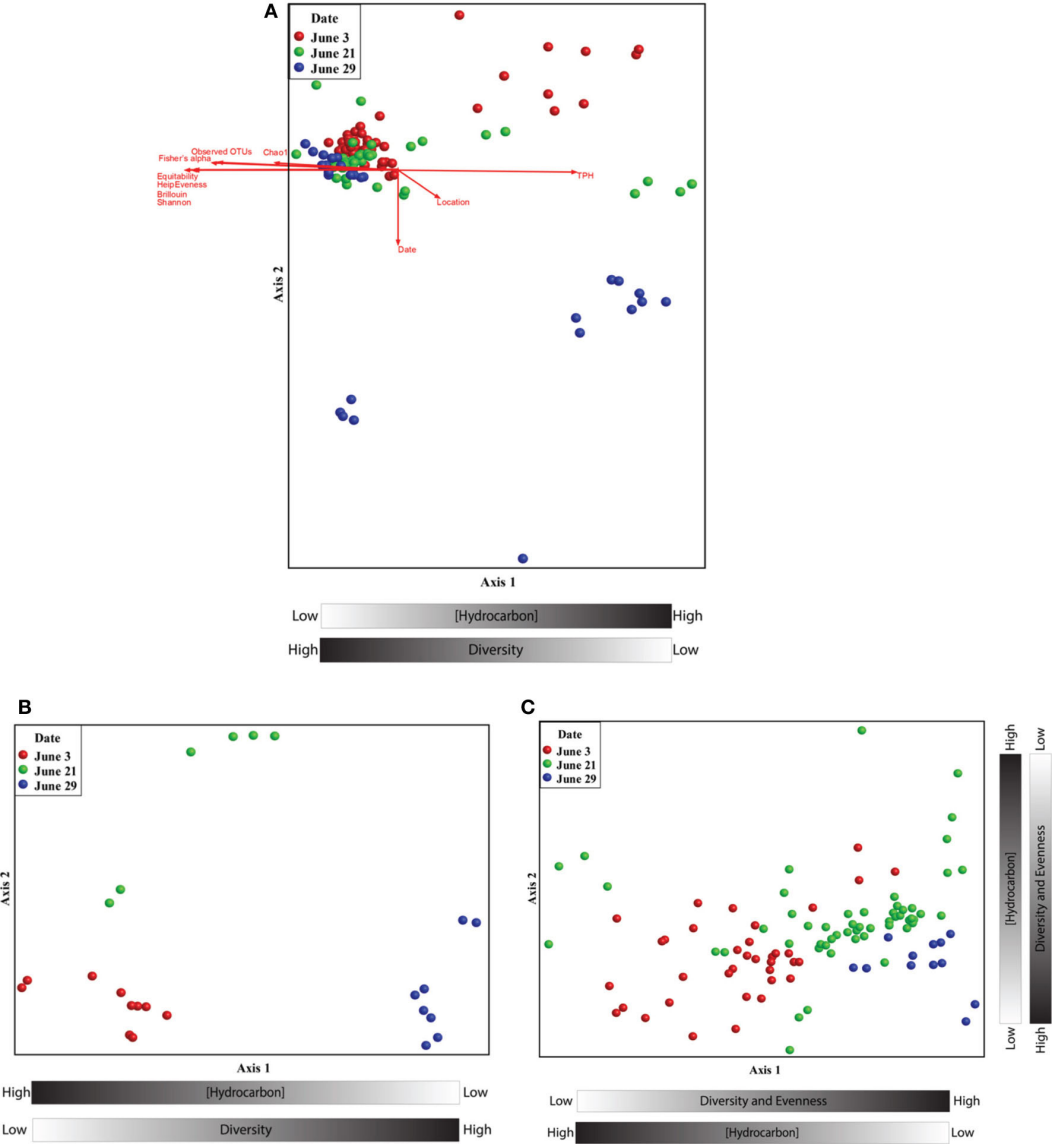
**(A)** Non-metric multidimensional scaling ordination of beached oil and sand samples based on the relative abundance pyrotag sequences assigned to family-level taxonomy. The ordination plot was rotated to maximize the degree of correlation with the total petroleum hydrocarbon variable. A two dimensional solution was found and the final stress was 0.023. **(B)** Non-metric multidimensional scaling ordinations of beached oil based on the relative abundance pyrotag sequences assigned to family-level taxonomy. The ordination plot was rotated to maximize the degree of correlation with the time variable. A two dimensional solution was found and the final stress was 0.039. **(C)** Non-metric multidimensional scaling ordinations of sand samples based on the relative abundance pyrotag sequences assigned to family-level taxonomy. The ordination plot was rotated to maximize the degree of correlation with the time variable. A two dimensional solution was found and the final stress was 0.086.

Non-metric multidimensional scaling (nMDS) analysis revealed a pronounced response of the microbial community to oil contamination (Figure 2). Samples with high TPH concentrations clustered separately from less contaminated samples (Pearson correlation to Axis 1; *r* = 0.971). In addition, the TPH concentration was inversely related to several alpha diversity measures (Figure 2 and Supplemental Figure S3). Co-inertia analyses revealed that the microbial communities differed significantly between the two types of contaminated samples: beached oil and oil-contaminated sand (*p*-value < 0.001). The beached-oil samples also clustered separately by time (Pearson correlation to Axis 1; *r* = 0.869), suggesting temporal shifts in the microbial community as a response to the oil spill (Figure 2B and Supplemental Table S2). The depletion in TPH was also positively correlated with time of sampling for all of the contaminated samples. Shifts in the microbial community aligned with continuous disappearance of hydrocarbons during the sampling period (Supplemental Table S2 and Table S3). Several PAHs and aliphatic hydrocarbon components were among the highest factors that correlated to Axis 2 on the nMDS plots (Figure 2C and Supplemental Table S3). Pearson correlations revealed that *Rhodobacteraceae* and *Alteromonadaceae* were most highly correlated with hydrocarbon concentrations in the contaminated samples (Supplemental Table S4) with genus and species-specific differences within sand and beached oil matrices. For example, sequences with closest homology to *Rhodobacter* sp., *Jannachia* sp., and *Marinobacter lutaoensis* had the highest correlation to beached oil samples (Supplemental Table S5), while *Ruegeria* sp., *Jannachia* sp., Alishewanella baltica, and *Pseudomonas pachastrellae* correlated with contaminated sand samples (Supplemental Table S6). Of these highest correlating OTUs, the *Marinobacter and Pseudomonas* genera were the most prevalent and abundant OTUs in the dataset, comprising up to 7 and 4% of the total community, respectively. It should also be noted, that microbial community composition and hydrocarbon profiles were highly correlated (Mantel test; *t* > 0, *p* = 0.00000, *r* = 0.6104).

### Metatranscriptomics of oil contaminated samples

In order to assess which hydrocarbon degradation genes were expressed, we studied the metatranscriptomic profiles of representative heavily oiled samples. Approximately 380 million paired end sequences (57 Gb) were retrieved from three beached oil samples, one from each sampling date (June 3, June 21, June 29). Our goal was to determine what types of genes were expressed in the beach community as a whole in response to heavy oil contamination. We found that 40–67% of the quality filtered reads contained ribosomal RNA genes, which was not surprising considering rRNA depletion was not applied to these samples prior to sequencing, given the low RNA yields. When analyzing which taxa were most prevalent in the rRNA from the metatranscriptomes, we saw similar trends to the 16S rRNA microbial community analysis. Metatranscriptome data matching the Greengenes SSU database were dominated by the proteobacteria (74%), more specifically the *Alteromonadales* (30%), *Oceanospirillales* (11%), and the *Rhodobacterales* (8%). Further, we found that when the metatrascriptome data were compared to the SSU Greengenes database, 19.2% of sequences annotated at the genus level matched to *Marinobacter*.

Even though the samples were dominated by ribosomal genes, more than 100 million of the quality filtered reads were available for functional gene annotation. Nearly 17 million of these reads matched to the hydrocarbon gene database. A total of 3553 different matches to the hydrocarbon database were retrieved from the metatranscriptomics data with an average of 2357 reads mapping to each hit. Comparison of the unassembled data to the hydrocarbon gene database revealed that enzymes involved in degradation of a variety of hydrocarbons, including PAHs were expressed; including a variety of monoxygenases and dioxygenases, and those involved in converting PAHs to dihydrodiols (Supplemental Table S7). Genes involved in the pathway for gentisate and substituted gentisate degradation were also expressed. Gentisate is a central metabolite in the aerobic biodegradation of both simple and complex aromatic hydrocarbons.

**Figure 3:**
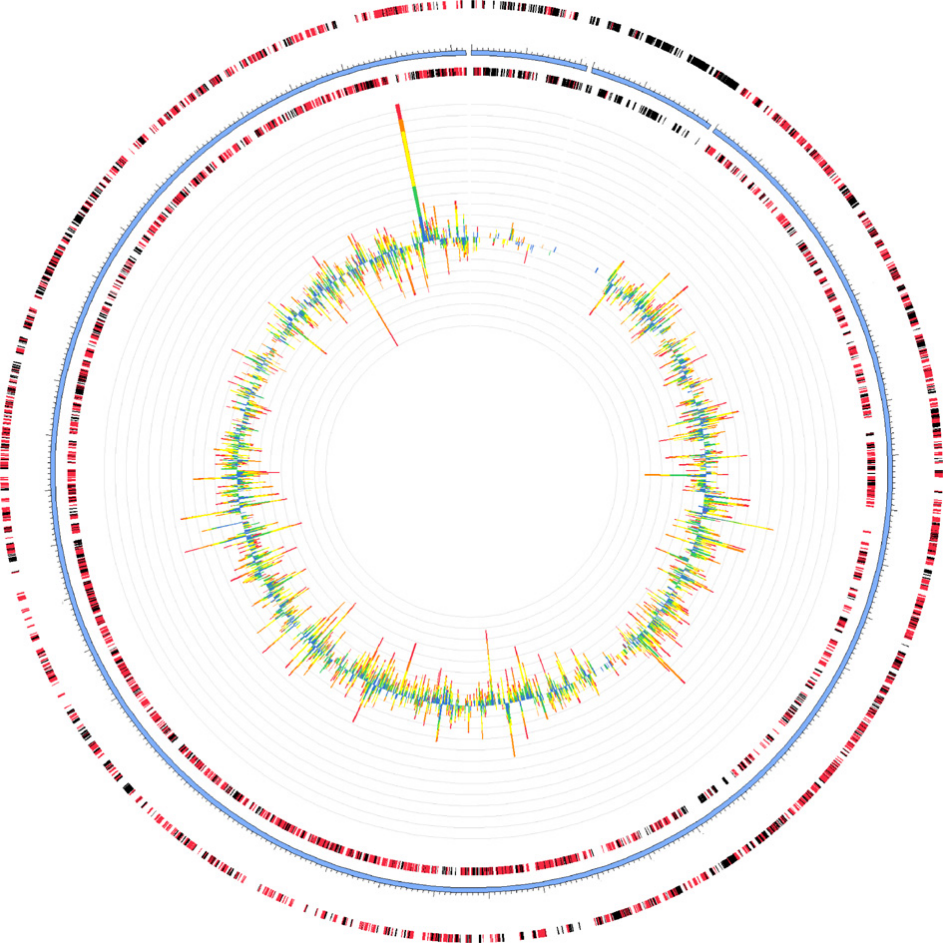
**Recruitment of June 3 metatranscriptome to *Marinobacter aquaeolei* strain VT8, the organism to which the largest number of contigs mapped for both metatranscriptomes**. The genome is approximately 4.8 Mb in size and the leading and lagging strands are represented by the outer most rings, separated by the blue ring, which indicates the position within the genome. Metatranscriptomic features are depicted as bar graphs inside the genome and their hit distribution is color-coded by e-value exponent as: blue, −3 to −5; green, −5 to −10; yellow, −10 to −20; orange, −20 to −30; red, less than −30. Figure was generated using the MG-RAST recruitment plot tool.

Two of the metatranscriptomes were assembled (those from the June 3 and June 21 sampling dates) yielding approximately 350,000 and 150,000 contigs (>150 bp), respectively, (Supplemental Table S8) and the assemblies were also screened for hydrocarbon degradation genes. When the metatranscriptomes were searched for matches to reference genomes in the MG-RAST database, *Marinobacter aquaeolei* strain VT8 was the closest match (94% average identity) (Figure 3). The most abundant xenobiotic degradation transcripts and overall functional transcripts matching to this strain were cyclohexanone monooxygenase, naphthyl-2-methylsuccinyl-CoA dehydrogenase, naphthyl-2-methylsuccinyl-CoA dehydrogenase, 3-hydroxyacyl-CoA dehydrogenase/ enoyl-CoA hydratase, and a succinate dehydrogenase complex (Table 1). Genes involved in motility were amongst the most abundant features of all contigs mapping to *M. aquaeolei* and included the CheA signal transduction histidine kinase involved in chemotaxis signaling and a flagellar hook-associated 2 domain-containing protein (Table 1).

**Table 1:** **Top xenobiotic and overall metatranscriptomic functions mapping to *Marinobacter aquaeolei***.

**Function**	Relative abundance June 3^*^	Relative abundance June 21^*^
**XENOBIOTIC**		
Cyclohexanone monooxygenase	0.382	0.114
Naphthyl-2-methylsuccinyl-CoA dehydrogenase	0.318	0.795
Glutathione S-transferase	0.255	0.000
3-hydroxyacyl-CoA dehydrogenase / enoyl-CoA hydratase	0.191	0.568
Succinate dehydrogenase	0.085	0.455
**OVERALL**		
CheA signal transduction histidine kinase	0.806	0.450
Flagellar hook-associated 2 domain-containing protein	0.467	0.340
Elongation factor Tu	0.042	1.023
Tetratricopeptide TPR_4	0.361	0.909

* *Relative abundances are percentages of total reads mapping to Marinobacter aquaelei*.

**Figure 4:**
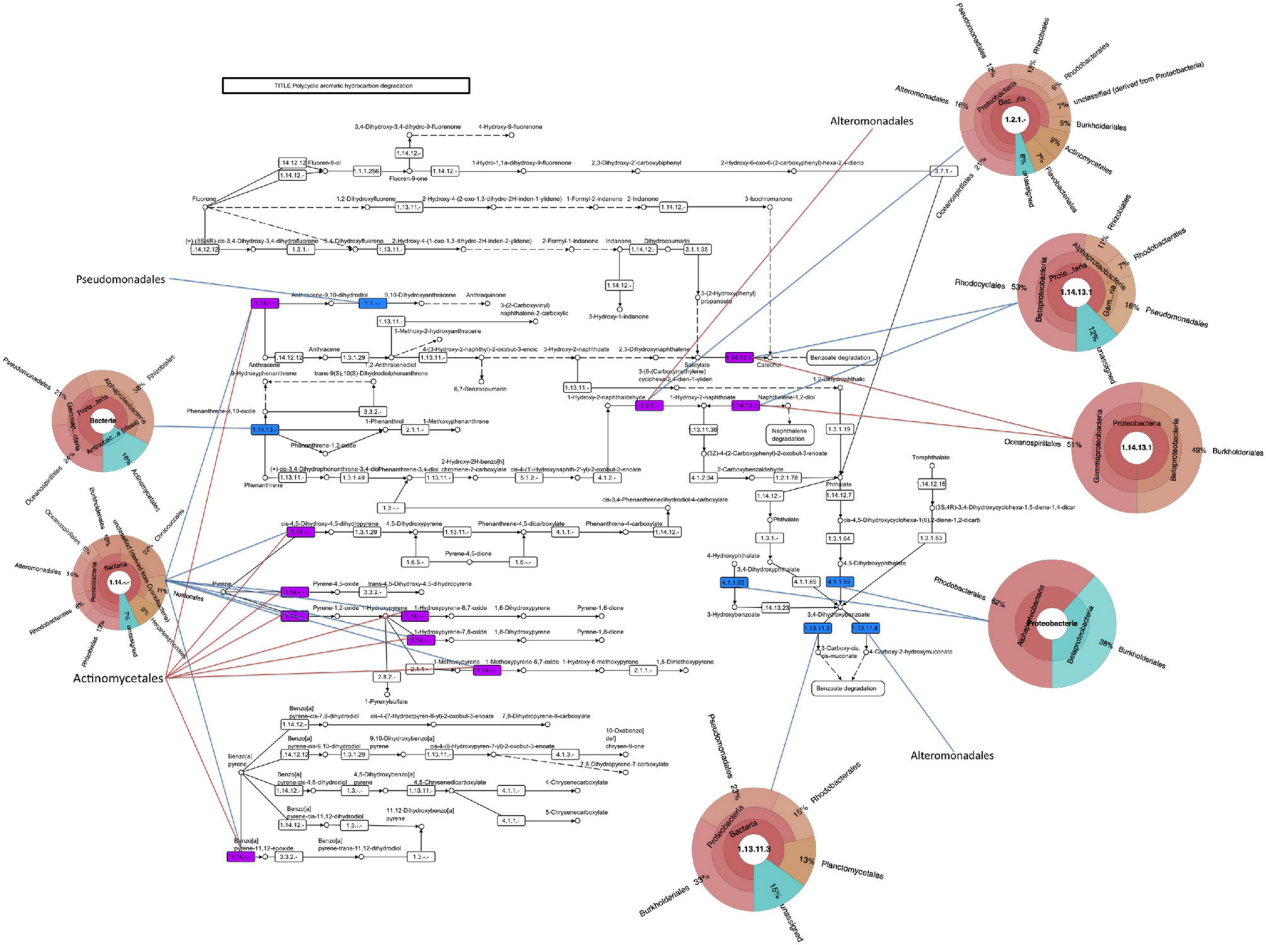
**Polycyclic aromatic hydrocarbon degradation pathway**. Assembled contigs are mapped to pathway from the KEGG database and colored in blue for June 3, red for June 21, and purple for presence in both time points. Pie charts indicating the best-hit taxonomic classification for each function were generated in Krona.

Besides *M. aquaeolei*, xenobiotic degradation transcripts mapped to several other *Proteobacteria* isolates in the MG-RAST database. For example, transcripts matched to PAH (Figure 4), *n*-alkane (Supplementary Figure S4A), and toluene degradation genes, matching to sequenced organisms in the *Pseudomonadales*, *Burkholderiales*, and *Alteromonadales*. Additionally, PAH (Figure 4), toluene, and benzoate pathways (Supplemental Figure S4B) mapped to members of the *Rhodobacterales* and toluene and benzoate metabolism transcripts mapped to *Rhizobiales*. It should be noted, that comparing metatranscriptomic data to KEGG pathways and organisms does not ascribe a complete pathway to a particular organism.

### Isolation of oil degrading strains from the contaminated beach samples

Enrichment with oil-contaminated samples from the sampling location resulted in isolation of 18 unique bacterial strains belonging almost entirely to the *Gammaproteobacteria*. 16S rRNA gene sequencing revealed that almost half of the isolates shared highest sequence homology to members of the *Pseudomonadales*, including *Pseudomonas stutzeri*, *Pseudomonas pachastrellae*, and *Pseudomonas alcaligenes* (Table 2). Three isolates belonging to the *Marinobacter* genus were retrieved from the more contaminated samples. Isolates having >99% sequence homology to known *Alcanivorax*, *Vibrio*, *Rheinheimera*, and *Bacillus* sp. were also retrieved from these samples. Most of the isolates were halophilic, Gram-negative organisms, and showed the potential for degrading the MC252 oil.

**Table 2:** **Cultured Isolates retrieved from beached oil and contaminated beach sands**.

**Isolate number**	**Sample source**	**Phylogenetic order**	**Closest relative in greengenes 16S rRNA gene database (accession no)**	**Similarity (%)**
2	Sand	Vibrionales	*Vibrio* sp. str. QY102 (AY174868.1)	99.86
3	Sand	Pseudomonadales	*Pseudomonas* sp. MOLA 58 (AM990833.1)	99.64
4	Sand	Vibrionales	*Vibrio* sp. str. QY102 (AY174868.1)	99.79
8	Sand	Vibrionales	*Vibrio* sp. str. QY102 (AY174868.1)	99.79
11	Sand	Alteromondales	Marinobacter sp. str. Libra (AY734434.1) or *Marinobacter hydrocarbonoclasticus* str. JL795 (EF512720.1)	99.93
12	Sand	Pseudomonadales	*Pseudomonas pachastrellae* str. PTG4-14 (EU603457.1)	97.14
14	Sand	Pseudomonadales	*Pseudomonas* sp. Da2 (AY570696.1)	99.43
16	Sand	Pseudomonadales	*Pseudomonas stutzeri* str. A1501 (NC_009434.1)	100
18	Sand	Bacillales	*Bacillus* sp. str. NRRL B-14911 (AAOX01000059.1)	99.72
19	Sand	Pseudomonadales	*Pseudomonas pseudoalcaligenes* str. 14 (AB276371.1)	99.86
23	Sand	Chromatiales	*Rheinheimera* sp. 97(2010) str. 97 (HM059656.1)	99.64
25	Sand	Alteromondales	*Marinobacter* sp. str. Libra (AY734434.1) or *Marinobacter hydrocarbonoclasticus* str. JL795 (EF512720.1)	99.79
26	Sand	Pseudomonadales	*Pseudomonas pseudoalcaligenes* str. 14 (AB276371.1)	98.58
31	Beached oil	Oceanospirillales	*Alcanivorax* sp. str. Abu-1 (AB053129.1)	99.64
32	Beached oil	Pseudomonadales	*Pseudomonas* sp. str. BJQ-B3 (FJ600357.1)	94.52
33	Beached oil	Alteromondales	*Marinobacter* sp. Str. NT N31 (AB166980.1)	98.32
35	Beached oil	Rhodobacterales	*Citreicella thiooxidans* str. 2PR57-8 (EU440958.1)	99.77
36	Beached oil	Rhodobacterales	*Roseobacter* sp. str. 49Xb1 (EU090129.1)	99.93

**Figure 5:**
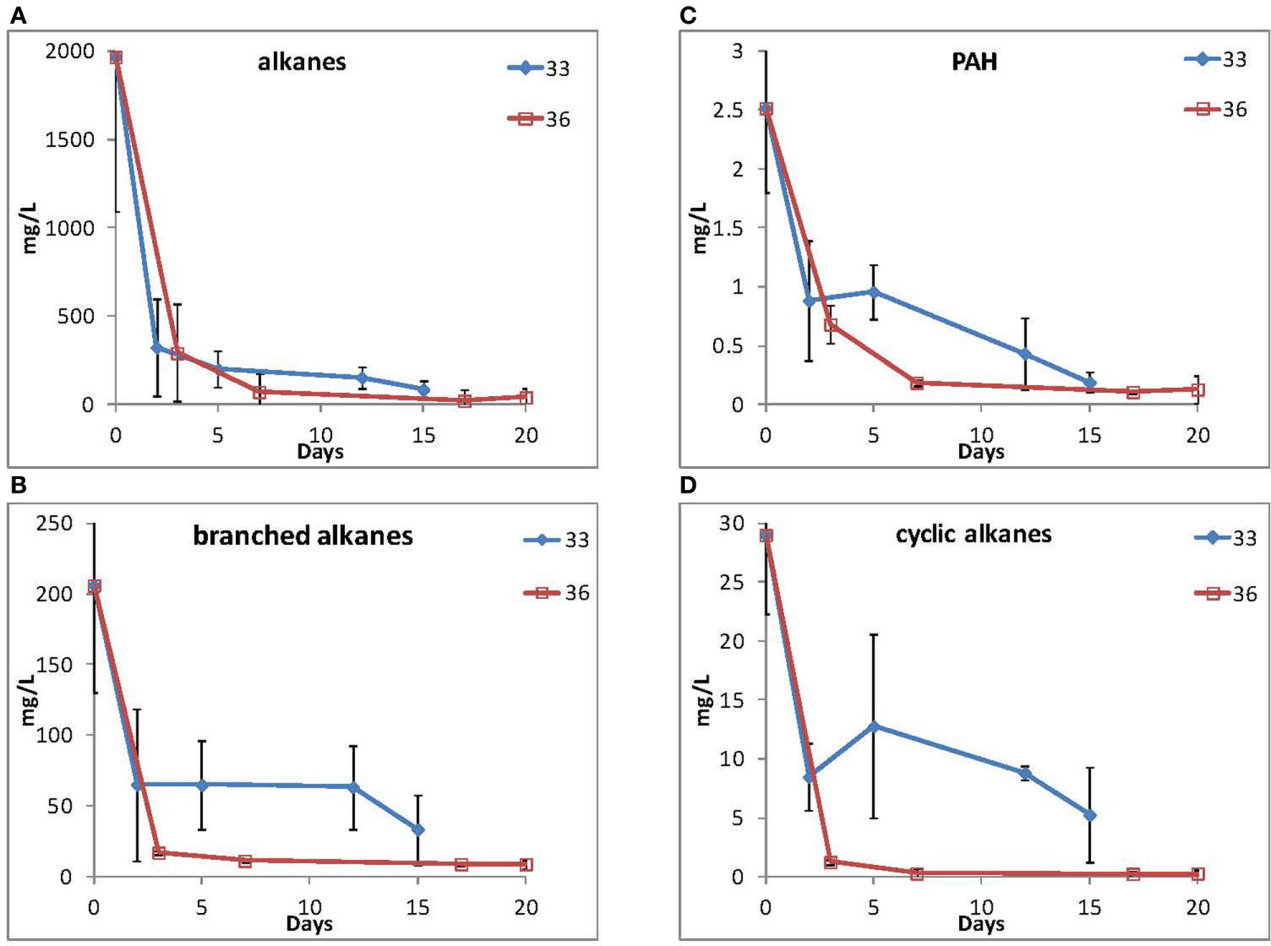
**Loss of **(A)** straight alkanes, **(B)** branched alkanes, **(C)** PAHs, and **(D)** cyclic alkanes during incubation by isolate 33 (*Marinobacter spp*.) and isolate 36 (*Roseobacter spp*.)**.

Because of their high relative abundance in the 16S rRNA gene data in contaminated samples, two representative isolates, 33 (*Marinobacter* spp.) and 36 (*Roseobacter* spp.) were selected for their ability to grow using MC252 as the carbon source. Total hydrocarbons were extracted at selected time points and straight chain alkanes and PAHs (Figure 5) were depleted during the incubations for both cultures, although longer alkanes (C25 and longer) persisted after 15 and 20 days of incubation, respectively (Supplemental Figure S5). It should be noted that the MC252 source oil used in the incubations was already depleted in the lighter hydrocarbons at the start of the incubation (Supplemental Figure S6).

## Discussion

Macondo oil from the *Deepwater Horizon* oil spill that reached the shore of Elmer's Beach caused shifts in the indigenous microbial communities in the beach sand toward a hydrocarbon-degrading consortium. This observation is consistent with previous studies that have assessed the impact of oil spills on coastline microbial communities (Kasai et al., 2001; Maruyama et al., 2003; Medina-Bellver et al., 2005; Alonso-Gutiérrez et al., 2009; Vila et al., 2010; Kostka et al., 2011). Kostka et al. (2011) also reported that highly contaminated samples exhibited higher bacterial cell densities than uncontaminated samples, and that there was a significant reduction in bacterial diversity associated with oil contamination. Here, we found that the contaminated samples collected from Elmer's Beach were generally dominated by *Alpha*- and *Gammaproteobacteria* (Figure 1) with up to 60% of the total microbial community being members of the *Alphaproteobacteria*. Other studies in the water column similarly reported a short-term shift of microbial communities toward specific members of the *Gammaproteobacteria* as an immediate response to crude oil inputs, which were then succeeded within 1 month by members of the *Alphaproteobacteria* (Abed et al., 2002; Röling et al., 2002; Hernandez-Raquet et al., 2006; Hazen et al., 2010; Redmond and Valentine, 2011; Valentine et al., 2012; Dubinsky et al., 2013).

Microbial community analysis revealed increases in the abundance of the *Rhodobacteraceae* and *Alteromonadaceae* in both the beached surface oil and contaminated beach sand samples. Therefore the different contaminated samples collected from the beach shared a similar bacterial community composition at the family level and exhibited parallel temporal successional changes in bacterial community structures driven by hydrocarbon inputs. During the first two sampling points, members of the *Alteromonadaceae*, with high sequence identity to *Marinobacter lutaoensis*, were very abundant in samples with high TPH concentrations. Members of the *Marinobacter* genus have previously been shown to be capable of degradation of both alkanes and PAH compounds with some isolates growing on single PAHs as their sole carbon source (Huu et al., 1999; Cohen, 2002; Shieh et al., 2003; Nicholson and Fathepure, 2004; Gerdes et al., 2005; Márquez and Ventosa, 2005; Brito et al., 2006; Gu et al., 2007; Cui et al., 2008; Rosano-Hernández and Fernández-Linares, 2009; Vila et al., 2010; Wu et al., 2010; Dos Santos et al., 2011). Here we also successfully isolated *Marinobacter* strains from contaminated beach samples, which were capable of growth on MC252 oil as their sole carbon source. Several previous studies have reported the role of *Marinobacter* in degradation of oil (Gerdes et al., 2005; Vila et al., 2010; Kostka et al., 2011). The potential biodegradation of oil by these isolates at ambient temperature further supports their potential for natural biodegradation of oil *in situ* (Figure 5). However, it should be noted that further work is needed to determine the exact nature of the hydrocarbon transformations that occurred during the incubations and whether they were mineralized or transformed to other metabolites.

Several bacterial taxa within the *Rhodobacteraceae* were abundant in the highly contaminated samples. The *Rhodobacteraceae* are metabolically and ecologically diverse, comprised of photoheterotrophs that can grow, either, photoautotropically or chemotrophically, as well as chemoorganotrophs, fermenters, and methylotrophs. Several members of the *Rhodobacteraceae* have previously been identified in oil polluted soils and marine environments and in fact have been shown to dominate oil polluted environments of the North Sea (Brakstad and Lødeng, 2005) and Southeast Asia (Harwati et al., 2008, 2009a,b). A few studies have demonstrated that the addition of photosynthetic bacteria to oil-polluted wastewater and soil triggers an increase in the abundance of hydrocarbon-oxidizing bacteria and thus enhances the rate of oil degradation (Martínez-Alonso et al., 2004; Llirós et al., 2008). Additionally, our cultivation-based experiments revealed that one representative of the *Rhodobacteraceae*, *Roseobacter* isolate 36, was also able to grow on MC252 as its sole carbon source. Overall, our data suggested oil degradation on the surface of beach sand that is exposed to light may have been promoted naturally by increases in photosynthetic populations.

Additionally many *Pseudomonas* species, having highest sequence homology to *P. pachastrellae*, were abundant in our 16S rRNA gene and cultivation experiments. Incidentally, similar pseudomonas strains were enriched from beach sands in the aftermath of both the Prestige oil spill in Northwestern Spain (Mulet et al., 2011) and other contaminated coastal sites during the Deepwater Horizon spill (Kostka et al., 2011) and these strains were shown to be central to the biodegradation of both aliphatic and aromatic hydrocarbons. Additionally, members of the *Alcanivorax* were abundant in the oil contaminated samples, corroborating previous 16S rRNA-based studies (Kasai et al., 2002; Kostka et al., 2011; Chakraborty et al., 2012).

Metatranscriptome analyses revealed that members of the *Alpha*- and *Gammaproteobacteria* were active in hydrocarbon degradation. This is the first study to determine functional genes involved in hydrocarbon degradation that were expressed in beach samples during the Deepwater Horizon spill. This study highlighted that metatranscriptomic data mapped to hydrocarbon degrading genes, including those involved in PAH, benzoate, and *n*-alkane degradation from *Alteromonadales*, *Pseudomonales*, and *Rhodobacterales* genomes. Data also mapped to other hydrocarbon degradation genes, including monooxygenases, dioxygenases, dehydrogenases, and hydratases, from members of these microbial classes. While this analysis doesn't necessarily ascribe a complete pathway to a particular organism, these results suggest that not only are these microorganisms abundant in the beach microbial community as suggested by the 16S rRNA gene data, but they may also play an active role in hydrocarbon degradation.

*Marinobacter aquaeolei* strain VT8 was the bacterium in the reference genome database that had the highest abundance of expressed genes in the oil contaminated samples, including those for cyclohexanone monooxygenase and naphthyl-2-methylsuccinyl-CoA dehydrogenase. In addition, transcripts for genes involved in chemotaxis and cellular motility mapped to *Marinobacter* suggesting that there was an active response to the hydrocarbon contamination in the beach communities, similar to the response observed for *Oceanospirillales* that were detected in the deep-sea plume (Mason et al., 2012). The high levels of gene expression observed for *Marinobacter* in the beach metatranscriptome data was supported by the finding that members of this genus were also enriched in the 16S rRNA data. In addition, we successfully isolated a representative of *Marinobacter* from the contaminated beach samples and demonstrated the ability of the isolate to degrade MC252 oil. These data suggest that *Marinobacter* may have played a key role in degradation of the oil that reached the coast during the Deepwater Horizon oil spill.

## Conclusions

During the Deepwater Horizon oil spill, MC252 oil originating from the Macondo well reached the coastline and Elmer's Beach was heavily impacted by the oil in June 2010, during which time we collected samples. Oil deposited on the shore appeared to cause a shift in the community structure toward a hydrocarbonoclastic consortia, as 16S rRNA gene sequencing demonstrated a diverse array of known petroleum hydrocarbon degrading microorganisms in these samples. Interestingly, several OTUs representative of previously described oil-degrading phototrophs were abundant in the heavily oiled samples from the first two sampling periods and these were succeeded by a diverse array of other potential oil-degrading bacteria. Metatranscriptome profiling revealed that members of the *Alpha*- and *Gammaproteobacteria* expressed genes for hydrocarbon degradation in the contaminated samples, suggesting that they played a key role in potential degradation processes. Of note, *Marinobacter* were abundant members of the community in the oil-contaminated samples and expressed genes for degradation of hydrocarbons. Compared to other oil spills that have impacted shorelines, such as the *Prestige* oil spill that occurred in a cold pristine habitat, the disappearance of MC252 oil seemed more rapid. This difference in microbial response could be due to differences in temperatures between the two sites as well as differences in other environmental variables, including previous exposure to oil spills. Overall, this study of the microbial community response on the Gulf shoreline may assist in the understanding of microbial proxies for oil contamination in similar coastal ecosystems.

## Author contributions

Regina Lamendella, Janet K. Jansson, and Terry C. Hazen were responsible for study conception and design. Regina Lamendella, Steven Strutt, and Janet K. Jansson were responsible for manuscript preparation. Sharon Borglin was responsible for chemical analyses. Romy Chakraborty was responsible for cultivation experiments. Regina Lamendella and Jenni Hultman were responsible for 16S RNA gene and metatranscriptomics experiments. Regina Lamendella, Steven Strutt, Olivia U. Mason, Emmanuel Prestat, and Neslihan Tas were responsible for bioinformatics and biostatistical analyses.

### Conflict of interest statement

The authors declare that the research was conducted in the absence of any commercial or financial relationships that could be construed as a potential conflict of interest.
