# Supplementary material for: Assessment of the Deepwater Horizon oil spill impact on Gulf coast microbial communities
Source: Front Microbiol. 2014 Apr 3;5:130. doi: 10.3389/fmicb.2014.00130 (PMC3982105; doi:10.3389/fmicb.2014.00130)
Supplement: Supplementary file 1 [file DataSheet1.DOCX]

Supplemental Tables

**Table S1.** Environmental parameters collected from Elmer’s Beach samples

| **Sample Name** | **Date** | **Location*(m)** | **TPH (mg/kg)** | **Core depth (cm)** | **Branched:aliphatic** | **C25:C17** |
| --- | --- | --- | --- | --- | --- | --- |
| EB_Foam01 | 6/29/2010 | 20 | 194.81 | 0 | 0.13 | 1.30 |
| EB_Foam02 | 6/29/2010 | 0 | 1160.88 | 0 | 0.14 | 0.63 |
| EB_Oil01 | 6/3/2010 | 0 | 789.40 | 0 | 0.15 | 0.57 |
| EB_Oil02 | 6/20/2010 | 1 | 10.88 | 0 | 0.22 | 0.71 |
| EB_Oil03 | 6/20/2010 | 1 | 1219.10 | 0 | 0.18 | 0.57 |
| EB_Oil04 | 6/20/2010 | 1 | 1507.78 | 0 | 0.15 | 0.58 |
| EB_Oil05 | 6/29/2010 | 20 | 638.63 | 0 | 0.16 | 0.52 |
| EB_Oil06 | 6/29/2010 | 20 | 1138.80 | 0 | 0.15 | 0.64 |
| EB_Water | 6/20/2010 | -1 | 6.54 | 0 | 0.15 | 0.57 |
| EB01_3cm | 6/3/2010 | 10 | 0.41 | 3 | 0.12 | 0.43 |
| EB01_6cm | 6/3/2010 | 10 | 0.45 | 6 | 0.16 | 0.37 |
| EB01_Surface | 6/3/2010 | 10 | 0.45 | 0 | 0.15 | 0.72 |
| EB02_3cm | 6/3/2010 | 10 | 0.12 | 3 | 0.18 | 0.51 |
| EB02_6cm | 6/3/2010 | 10 | 0.01 | 6 | 0.22 | 0.63 |
| EB02_9cm | 6/3/2010 | 10 | 0.00 | 9 | 0.14 | 0.45 |
| EB02_Surface | 6/3/2010 | 10 | 0.06 | 0 | 0.11 | 0.97 |
| EB03_3cm | 6/3/2010 | 0 | 5.70 | 3 | 0.19 | 0.73 |
| EB03_6cm | 6/3/2010 | 0 | 3.71 | 6 | 0.12 | 0.71 |
| EB03_9cm | 6/3/2010 | 0 | 0.38 | 9 | 0.15 | 2.14 |
| EB03_Oil | 6/3/2010 | 0 | 1085.31 | 0 | 0.14 | 0.60 |
| EB03_Surface | 6/3/2010 | 0 | 53.51 | 0 | 0.14 | 0.59 |
| EB04_3cm | 6/3/2010 | 5 | 9.24 | 3 | 0.15 | 0.66 |
| EB04_Surface | 6/3/2010 | 5 | 17.97 | 0 | 0.22 | 0.54 |
| EB05_3cm | 6/3/2010 | 10 | 23.87 | 3 | 0.14 | 0.70 |
| EB05_6cm | 6/3/2010 | 10 | 0.84 | 9 | 0.13 | 0.51 |
| EB05_9cm | 6/3/2010 | 10 | 0.84 | 9 | 0.13 | 0.51 |
| EB05_Surface | 6/3/2010 | 10 | 18.61 | 0 | 0.14 | 0.62 |
| EB05_Oil | 6/3/2010 | 5 | 1113.88 | 0 | 0.12 | 0.05 |
| EB05_Surface | 6/3/2010 | 10 | 1443.72 | 0 | 0.13 | 0.70 |
| EB06_3cm | 6/3/2010 | 10 | 0.41 | 3 | 0.17 | 0.64 |
| EB06_6cm | 6/3/2010 | 10 | 8.37 | 6 | 0.17 | 0.80 |
| EB06_9cm | 6/3/2010 | 10 | 0.75 | 9 | 0.44 | 1.11 |
| EB06_Oil | 6/3/2010 | 10 | 916.99 | 0 | 0.15 | 0.04 |
| EB06_Surface | 6/3/2010 | 10 | 303.84 | 0 | 0.15 | 0.45 |
| EB07_3cm | 6/3/2010 | 10 | 17.40 | 3 | 0.15 | 0.40 |
| EB07_6cm | 6/3/2010 | 10 | 2.13 | 6 | 0.12 | 0.57 |
| EB07_9cm | 6/3/2010 | 10 | 0.38 | 9 | 0.17 | 0.56 |
| EB07_Oil | 6/3/2010 | 10 | 2071.87 | 0 | 0.10 | 0.88 |
| EB07_Surface | 6/3/2010 | 10 | 13.45 | 0 | 0.16 | 1.48 |
| EB08_3cm | 6/20/2010 | 1 | 0.04 | 3 | 0.18 | 1.36 |
| EB08_6cm | 6/20/2010 | 1 | 0.08 | 6 | 0.15 | 1.28 |
| EB08_9cm | 6/20/2010 | 1 | 0.03 | 9 | 0.29 | 1.07 |
| EB08_Surface | 6/20/2010 | 1 | 0.07 | 0 | 0.18 | 0.82 |
| EB09_3cm | 6/20/2010 | 1 | 0.09 | 3 | 0.13 | 1.78 |
| EB09_6cm | 6/20/2010 | 1 | 0.03 | 6 | 0.20 | 1.30 |
| EB09_9cm | 6/20/2010 | 1 | 0.14 | 9 | 0.54 | 0.49 |
| EB09_Surface | 6/20/2010 | 1 | 0.05 | 0 | 0.10 | 1.64 |
| EB10_3cm | 6/20/2010 | 1 | 0.01 | 3 | 0.24 | 1.10 |
| EB10_6cm | 6/20/2010 | 1 | 0.35 | 6 | 0.21 | 0.91 |
| EB10_9cm | 6/20/2010 | 1 | 0.00 | 9 | 0.21 | 1.38 |
| EB10_Surface | 6/20/2010 | 1 | 0.00 | 0 | 0.13 | 0.64 |
| EB11_3cm | 6/20/2010 | 2 | 0.01 | 3 | 0.27 | 1.28 |
| EB11_6cm | 6/20/2010 | 2 | 0.00 | 6 | 0.23 | 0.38 |
| EB11_9cm | 6/20/2010 | 2 | 0.05 | 9 | 0.37 | 0.95 |
| EB11_Surface | 6/20/2010 | 2 | 0.02 | 0 | 0.19 | 1.95 |
| EB12_3cm | 6/20/2010 | 2 | 0.00 | 3 | 0.23 | 1.17 |
| EB12_6cm | 6/20/2010 | 2 | 0.04 | 6 | 0.23 | 1.99 |
| EB12_9cm | 6/20/2010 | 2 | 0.09 | 9 | 0.20 | 3.14 |
| EB12_Surface | 6/20/2010 | 2 | 16.90 | 0 | 0.03 | 0.01 |
| EB13_3cm | 6/20/2010 | 0 | 0.07 | 3 | 0.34 | 4.16 |
| EB13_6cm | 6/20/2010 | 0 | 0.12 | 6 | 0.24 | 0.85 |
| EB13_9cm | 6/20/2010 | 0 | 2.20 | 9 | 0.17 | 0.73 |
| EB13_Surface | 6/20/2010 | 0 | 0.00 | 0 | 0.29 | 0.83 |
| EB14_3cm | 6/20/2010 | -1 | 53.23 | 3 | 0.16 | 0.56 |
| EB14_6cm | 6/20/2010 | -1 | 75.11 | 6 | 0.15 | 0.52 |
| EB14_9cm | 6/20/2010 | -1 | 44.17 | 9 | 0.15 | 0.58 |
| EB14_Surface | 6/20/2010 | -1 | 51.62 | 0 | 0.16 | 0.63 |
| EB15_3cm | 6/29/2010 | 10 | 3.09 | 3 | 0.07 | 9.09 |
| EB15_6cm | 6/29/2010 | 10 | 0.42 | 6 | 0.00 | 1.38 |
| EB15_9cm | 6/29/2010 | 10 | 0.21 | 9 | 0.00 | 0.00 |
| EB15_Surface | 6/29/2010 | 10 | 25.12 | 0 | 0.17 | 1.23 |
| EB16_3cm | 6/29/2010 | 0 | 0.23 | 3 | 0.43 | 1.05 |
| EB16_6cm | 6/29/2010 | 0 | 0.29 | 6 | 0.66 | 1.04 |
| EB16_9cm | 6/29/2010 | 0 | 0.40 | 9 | 0.30 | 1.72 |
| EB16_Surface | 6/29/2010 | 0 | 2.22 | 0 | 0.02 | 77.54 |
| EB17_3cm | 6/29/2010 | -1 | 0.43 | 3 | 0.36 | 1.64 |
| EB17_6cm | 6/29/2010 | -1 | 0.22 | 6 | 0.00 | 0.52 |
| EB17_Surface | 6/29/2010 | -1 | 0.32 | 0 | 0.18 | 1.76 |

*Location refers to distance perpendicular to waterline. Zero indicates samples taken at waterline, negative numbers indicate samples taken in the water while positive numbers are samples taken on the beach

**Table S2.** Pearson Correlations to Environmental Parameters for nMDS of beached oil samples

| **Environmental Parameter** | **Pearson Correlation Coefficient** | **Axis of Correlation** |
| --- | --- | --- |
| Heip Eveness | 0.73 | Axis 1 |
| Equitability | 0.722 | Axis 1 |
| Brillouin diversity | 0.694 | Axis 1 |
| Shannon | 0.687 | Axis 1 |
| Date | 0.602 | Axis 1 |
| Observed OTUs | 0.562 | Axis 1 |
| Fisher's alpha | 0.561 | Axis 1 |
| Doubletons | 0.554 | Axis 1 |
| Simpson | 0.474 | Axis 1 |
| Core Depth | 0.424 | Axis 1 |
| Singletons | 0.394 | Axis 1 |
| Chao 1 | 0.372 | Axis 1 |
| C25:C17 | 0.361 | Axis 1 |
| Branched:aliphatic | 0.249 | Axis 1 |
| Naphthalene, C5 | 0.472 | Axis 2 |
| Anthracene | 0.462 | Axis 2 |
| Phenanthrene | 0.456 | Axis 2 |
| Simpson_E | 0.446 | Axis 2 |
| Naphthalene, C2 | 0.444 | Axis 2 |
| Phenanthrene, C1 | 0.441 | Axis 2 |
| Phenanthrene, C2 | 0.432 | Axis 2 |
| Phenanthrene, C4 | 0.43 | Axis 2 |
| 9H-Fluorene, C2 | 0.424 | Axis 2 |
| Naphthalene, C3 | 0.423 | Axis 2 |
| 9H-Fluorene, C1 | 0.419 | Axis 2 |
| 9H-Fluorene, 2C2 | 0.413 | Axis 2 |
| PAH | 0.411 | Axis 2 |
| 9H Flourene, 2C1 | 0.41 | Axis 2 |
| Naphthalene, C4 | 0.408 | Axis 2 |
| Phenanthrene, C3 | 0.406 | Axis 2 |
| Phenanthrene, C3 | 0.406 | Axis 2 |
| Naphthalene, C1 | 0.406 | Axis 2 |
| Acenaphthylene | 0.396 | Axis 2 |
| Phenanthrene, 2C2 | 0.384 | Axis 2 |
| Phenanthrene, 2C3 | 0.382 | Axis 2 |
| Pyrene | 0.379 | Axis 2 |
| Phenanthrene, 2C1 | 0.377 | Axis 2 |
| Chrysene | 0.374 | Axis 2 |
| n-Pentacosane | 0.369 | Axis 2 |
| phytane (branched) | 0.368 | Axis 2 |
| n-Nonadecane | 0.366 | Axis 2 |
| tricosane | 0.365 | Axis 2 |
| Benz[a]anthracene | 0.362 | Axis 2 |
| pristane (branched) | 0.361 | Axis 2 |
| n-octadecane | 0.359 | Axis 2 |
| eicosane | 0.358 | Axis 2 |
| n-hexadecane | 0.337 | Axis 2 |
| n-heptadecane | 0.323 | Axis 2 |
| TPH | 0.286 | Axis 2 |
| Fisher's Alpha | -0.414 | Axis 2 |
| Observed OTUs | -0.425 | Axis 2 |
| Doubletons | -0.46 | Axis 2 |

**Table S3.** Pearson Correlations to Environmental Parameters for nMDS of sand samples

| **Environmental parameter** | **Pearson Correlation Coefficient** | **Axis of Correlation** |
| --- | --- | --- |
| Date | 0.869 | Axis 1 |
| Chao 1 | 0.619 | Axis 1 |
| Singletons | 0.548 | Axis 1 |
| Shannon | 0.538 | Axis 1 |
| brillouin_diversity | 0.537 | Axis 1 |
| Observed OTUs | 0.525 | Axis 1 |
| Equitability | 0.521 | Axis 1 |
| Fisher Alpha | 0.517 | Axis 1 |
| Heip Eveness | 0.502 | Axis 1 |
| Acenaphthylene | 0.482 | Axis 1 |
| Simpson | 0.465 | Axis 1 |
| Doubletons | 0.312 | Axis 1 |
| C25:C17 | 0.301 | Axis 1 |
| Naphthalene, C1 | -0.37 | Axis 1 |
| Phenanthrene, 2C1 | -0.379 | Axis 1 |
| Pyrene | -0.389 | Axis 1 |
| n-Pentacosane | -0.424 | Axis 1 |
| n-hexadecane | -0.463 | Axis 1 |
| Dominance | -0.465 | Axis 1 |
| McIntosh-E | -0.466 | Axis 1 |
| n-octadecane | -0.472 | Axis 1 |
| eicosane | -0.49 | Axis 1 |
| phytane (branched) | -0.496 | Axis 1 |
| n-Nonadecane | -0.5 | Axis 1 |
| tricosane | -0.506 | Axis 1 |
| pristane (branched) | -0.509 | Axis 1 |
| n-heptadecane | -0.514 | Axis 1 |
| Simpson evenness | -0.536 | Axis 1 |
| Branched:aliphatic | 0.482 | Axis 2 |
| Chao1 | 0.295 | Axis 2 |
| Singletons | 0.286 | Axis 2 |
| PAH | -0.317 | Axis 2 |
| Pyrene | -0.32 | Axis 2 |
| 9H Flourene, 2C1 | -0.32 | Axis 2 |
| Phenanthrene, C1 | -0.323 | Axis 2 |
| Phenanthrene, C3 | -0.325 | Axis 2 |
| Phenanthrene, 2C2 | -0.328 | Axis 2 |
| Benzo[a]pyrene | -0.328 | Axis 2 |
| Phenanthrene, C2 | -0.329 | Axis 2 |
| n-hexadecane | -0.348 | Axis 2 |
| Phenanthrene, 2C1 | -0.348 | Axis 2 |
| n-Pentacosane | -0.36 | Axis 2 |
| n-octadecane | -0.361 | Axis 2 |
| phytane (branched) | -0.386 | Axis 2 |
| pristane (branched) | -0.407 | Axis 2 |
| n-heptadecane | -0.413 | Axis 2 |
| eicosane | -0.415 | Axis 2 |
| n-Nonadecane | -0.418 | Axis 2 |
| tricosane | -0.426 | Axis 2 |

**Table S4.** Pearson Correlations to Total Petroleum Hydrocarbon Concentration in Beached Oil Samples

| **Lineage** | **Pearson Correlation Coefficient** | **Axis of Correlation** |
| --- | --- | --- |
| Bacteria;Proteobacteria;Alphaproteobacteria;Rhodobacterales;Rhodobacteraceae | -0.872 | Axis 1 |
| Bacteria;Proteobacteria;Gammaproteobacteria;Alteromondales;Alteromonadaceae | -0.819 | Axis 1 |
| Bacteria;Proteobacteria;Gammaproteobacteria;Chromatiaceae;Chromatiaceae | -0.794 | Axis 1 |
| Bacteria;Proteobacteria;Deltaproteobacteria;Desulfuomonadales;Geobacteraceae | -0.702 | Axis 1 |
| Bacteria;Bacteroidetes;Sphingobacteria;Sphingobacteriales;Flexibacteraceae | 0.705 | Axis 1 |
| Bacteria;Actinobacteria;Actinobacteria;Actinomycetales;Mycobacteriaceae | 0.708 | Axis 1 |
| Bacteria;Bacteroidetes;Sphingobacteria;Sphingobacteriales;Sphingobacteriaceae | 0.711 | Axis 1 |
| Bacteria;Proteobacteria;Gammaproteobacteria;Pasteurellales;Pasteurellaceae | 0.727 | Axis 1 |
| Bacteria;Actinobacteria;Actinobacteria;Actinomycetales;Corynebacterineae | 0.733 | Axis 1 |
| Bacteria;Actinobacteria;Actinobacteria;Actinomycetales;Propionibacterineae | 0.736 | Axis 1 |
| Bacteria;Proteobacteria;Alphaproteobacteria;Rhizobiales;Rhizobiaceae | 0.738 | Axis 1 |
| Bacteria;Chloroflexi;Anaerolineae;A4b;TK32 | 0.742 | Axis 1 |
| Bacteria;Proteobacteria;Alphaproteobacteria;Rhodospriallales;Acetobacteraceae | 0.746 | Axis 1 |
| Bacteria;Proteobacteria;Alphaproteobacteria;Rhodospriallales;Hyphomicrobiaceae | 0.767 | Axis 1 |
| Bacteria;Proteobacteria;Gammaproteobacteria;Xanthomondales;Xanthomonadaceae | 0.816 | Axis 1 |
| Bacteria;Planctomycetes;Planctomycetacia;Isospaerales;Isosphaeraceae | 0.822 | Axis 1 |
| Bacteria;Proteobacteria;Gammaproteobacteria;CCD24;JG30 | 0.861 | Axis 1 |
| Bacteria;Proteobacteria;Alphaproteobacteria;Sphingomonadales;Erythrobacteraceae | 0.864 | Axis 1 |
| Bacteria;Actinobacteria;Actinobacteria;Coriobacteriales;Coriobacterineae | 0.841 | Axis 2 |
| Bacteria;Proteobacteria;Deltaproteobacteria;Desulfuomonadales;Desulfovibrionaceae | 0.852 | Axis 2 |
| Bacteria;Firmicutes;Clostridia;Clostridiales;Acidaminococcaceae | 0.854 | Axis 2 |
| Bacteria;Bacteroidetes;Bacteroidales;Rikenellaceae;Rikenellaceae | 0.871 | Axis 2 |
| Bacteria;Firmicutes;Mollicutes;Clostridia;Hyphomicrobiaceae | 0.886 | Axis 2 |
| Bacteria;Firmicutes;Clostridia;Clostridiales;Clostridiaceae | 0.922 | Axis 2 |
| Bacteria;Bacteroidetes;Flavobacteriaceae;Flavobacteriales;Flavobacteriaceae | -0.755 | Axis 2 |

**Table S5.** OTUs correlating with beached oil and sand samples collected from Elmer’s Beach over three time points in June, 2010.

| **Closest Match** | **Percent Identity** | **Consensus Lineage** | **Samples** | **Pearson Correlation** |
| --- | --- | --- | --- | --- |
| marine macro-alga surface clone UA03 | 96.7 | Bacteria;Proteobacteri;Alphaproteobacteria | BO | -0.828 |
| Rhodobacter sp. str. EMB 174 | 94.48 | Bacteria;Proteobacteria;Alphaproteobacteria;Rhodobacterales;Rhodobacteraceae | BO | -0.822 |
| JCM 11179T | 100 | Bacteria;Proteobacteria;Gammaproteobacteria;Alteromonadales;Alteromonadaceae;Marinobacter | BO | -0.802 |
| Microbial Carbon and Nitrogen Cycling Sediments South Atlantic Bight Permeable Shelf Sediment clone LC1-19 | 97.78 | Bacteria;Proteobacteria;Deltaproteobacteria; | BO and sand | -0.792 |
| Thiorhodococcus minor str. CE2203 | 97.22 | Bacteria;Proteobacteria;Gammaproteobacteria;Chromatiales;Chromatiaceae | BO | -0.784 |
| Abundance and microbial life ocean crust seafloor lavas Hawai'i South Point X3 clone P0X3b1C12 | 95.58 | Bacteria;Nitrospirae;Thermodesulfovibrionales;Thermodesulfovibrionaceae; | BO | -0.762 |
| Facilitation robust growth colonies and dilute liquid s 'helper' heterotrophic Prochlorococcus SS120 isolate EZ49Rhodospirillaceae str. EZ49 | 100 | Bacteria;Proteobacteria;Alphaproteobacteria;Rhodospirillales;Rhodospirillaceae | BO | -0.751 |
| tallgrass prairie soil clone FFCH17833 | 95.12 | Bacteria;Proteobacteria;Alphaproteobacteria;Oleomonas | BO | -0.745 |
| Cultivating Sargasso Sea clone GMD21A06 | 100 | Bacteria;Proteobacteria;Alphaproteobacteria | BO | -0.741 |
| Mt. Nan-Jen litterfall clone IYF49 | 93.33 | Bacteria;Proteobacteria;Alphaproteobacteria | BO | -0.741 |
| Ruegeria sp. str. QDHT-11 | 96.67 | Bacteria;Proteobacteria;Alphaproteobacteria;Rhodobacterales;Rhodobacteraceae;Ruegeria | Sand | -0.74 |
| Cellulophaga sp. str. DS3 | 96.72 | Bacteria;Bacteroidetes;Flavobacteria;Flavobacteriales;Flavobacteriaceae;Cellulophaga | BO | -0.735 |
| sponge clone A10A5 | 97.22 | Archaea;Thaumarchaeota;Cenarchaeales;Cenarchaeum | BO | -0.735 |
| Jannaschia sp. str. ZXM109 | 100 | Bacteria;Proteobacteria;Alphaproteobacteria;Rhodobacterales | BO and sand | -0.704 |
| Alishewanella baltica str. BA131 | 95 | Bacteria;Proteobacteria;Gammaproteobacteria;Alteromonadales;Alteromonadaceae;Alishewanella | Sand | -0.672 |
| Microbial dynamics -arctic exposure hydrocarbons and bioremediation agent intertidal beach sediment clone SR-O-03-32 | 96.61 | Bacteria;Proteobacteria;Gammaproteobacteria;Moraxellaceae | Sand | -0.653 |
| Pseudomonas pachastrellae str. KMM 331 | 96.67 | Bacteria;Proteobacteria;Gammaproteobacteria;Pseudomonadales;Pseudomonadaceae;Pseudomonas | Sand | -0.64 |
| Cellulophaga sp. str. DS3 | 96.72 | Bacteria;Bacteroidetes;Flavobacteria;Flavobacteriales;Flavobacteriaceae;Cellulophaga | Sand | -0.635 |
| consecutively aerated submerged treating domestic wastewater activated sludge membrane bioreactor clone AS6 | 95.58 | Bacteria;Proteobacteria;Gammaproteobacteria;Betaproteobacteria;Comamonadaceae | Sand | -0.613 |
| lake microbial mat isolate str. R-9219 | 100 | Bacteria;Proteobacteria;Alphaproteobacteria | Sand | -0.59 |
| Comamonas sp. str. SS-1 | 100 | Bacteria;Proteobacteria;Betaproteobacteria;Burkholderiales;Comamonadaceae;Comamonas | Sand | -0.544 |
| mangrove soil clone MSB-4G8 | 97.78 | Bacteria;Proteobacteria;Deltaproteobacteria;Desulfobacteraceae | Sand | 0.479 |
| marine ANAMMOX sediment clone AY592135 | 98.67 | Bacteria;Gemmatimonadetes;Gemm_4 | Sand | 0.482 |
| Abundance and microbial life ocean crust seafloor lavas Hawai'i South Point X3 clone P0X3b5G09 | 96.15 | Bacteria;Acidobacteria | Sand | 0.495 |
| Guerrero Negro hypersaline microbial mat clone 05D2Z68 | 98.89 | Bacteria;Planctomycetes;Planctomycetacia | Sand | 0.5 |
| Xiamen China mangrove sediment clone XME38 | 95.95 | Bacteria;Chloroflexi;Anaerolineae;Anaerolineales | Sand | 0.51 |
| inactive deep-sea hydrothermal vent chimneys clone IheB3-34 | 97.16 | Bacteria;Actinobacteria;Acidimicrobidae | Sand | 0.566 |
| Nullarbor cave clone wb1_A18 | 96.67 | Bacteria;Gemmatimonadetes;Gemm-6 | Sand | 0.595 |
| microorganisms catalyzing nitrogen removal and organic matter metabolism permeable marine sediments Gulf Mexico sediment clone 3G02-06 | 98.89 | Bacteria;Proteobacteria;Deltaproteobacteria | Sand | 0.599 |
| harbor sediment clone VHS-B4-23 | 94.51 | Bacteria;Proteobacteria;Deltaproteobacteria;Desulfobacteraceae | Sand | 0.618 |
| Pontibacter actiniorum str. KMM 6156 | 99.44 | Bacteria;Proteobacteria;Alphaproteobacteria;Pontibacter | BO | 0.813 |
| Short-term effect organic matter on nitrite oxidation performance structure nitrite-oxidizing suspension composed Nitrobacter Nitrospira and heterotrophic Sanwayao wastewater plant (WWTP) clone 1-E | 100 | Bacteria;Firmicutes;Clostridia;Clostridiales;Clostridiaceae | BO | 0.818 |
| Clostridium ruminantium str. LA1 | 100 | Bacteria;Bacteroidetes;Flavobacteria;Flavobacteriales;Flavobacteriaceae;Clostridium | BO | 0.826 |
| Flavobacterium sp. str. WPCB080 | 99.44 | Bacteria;Proteobacteria;Alphaproteobacteria;Flavobacterium | BO | 0.828 |
| Isolation and physiological two piezophilic thermophilic chemolithoautotrophs deep-sea hydrothermal vent black smoker chimney isolate 108KT-2008 str. 108 | 100 | Bacteria;Proteobacteria;Alphaproteobacteria | BO | 0.839 |
| Hydrocarbon-Degrading Indonesian Seawater seawater isolate B51B51 str. B51 | 96.67 | Bacteria;Proteobacteria;Alphaproteobacteria;Sphingomonadales;Erythrobacteraceae | BO | 0.878 |
| Erythrobacter sp. str. K10-17 | 100 | Bacteria;Proteobacteria;Alphaproteobacteria;Erythrobacter | BO | 0.88 |

**Table S6.** Pearson Correlations to Total Petroleum Hydrocarbon Concentration in Sand Samples

| **Lineage** | **Pearson Correlation Coefficient** | **Axis of Correlation** |
| --- | --- | --- |
| Bacteria;Proteobacteria;Gammaproteobacteria;Alteromondales;Alteromonadaceae | -0.813 | Axis 1 |
| Bacteria;Bacteroidetes;Flavobacteriaceae;Flavobacteriales;Flavobacteriaceae | -0.75 | Axis 1 |
| Bacteria;Proteobacteria;Alphaproteobacteria;Rhodobacterales;Rhodobacteraceae | -0.691 | Axis 1 |
| Bacteria;Proteobacteria;Betaproteobacteria;Burkholderiales;Comamondaceae | -0.644 | Axis 1 |
| Bacteria;Bacteroidetes;Sphingobacteria;Sphingobacteriales;Flexibacteraceae | -0.592 | Axis 1 |
| Bacteria;Proteobacteria;Gammaproteobacteria;Pseudomondales;Pseudomonadaceae | -0.559 | Axis 1 |
| Bacteria;Proteobacteria;Alphaproteobacteria;Sphingomonadales;Erythrobacteraceae | -0.548 | Axis 1 |
| Bacteria;Proteobacteria;Gammaproteobacteria;Thiotrichales;Piscirickettsiaceae | -0.546 | Axis 1 |
| Eukaryota;Fungi;Ascomycota;Pezizomycotina | -0.543 | Axis 1 |
| Bacteria;Proteobacteria;Alphaproteobacteria;Sphingomonadales;Sphingomonadaceae | -0.452 | Axis 1 |
| Bacteria;Proteobacteria;Gammaproteobacteria;Xanthomondales;Xanthomonadaceae | -0.413 | Axis 1 |
| Bacteria;Proteobacteria;Betaproteobacteria;Burkholderiales;Burkholderiaceae | -0.381 | Axis 1 |
| Bacteria;Firmicutes;Mollicutes;Clostridia;Hyphomicrobiaceae | -0.375 | Axis 1 |
| Bacteria;Bacteria;Actinobacteria;Actinobacteria;Actinomycetales | -0.371 | Axis 1 |
| Bacteria;Bacteria;Proteobacteria;Gammaproteobacteria;Oceanospiralles;Alcanivoraceae | -0.367 | Axis 1 |
| Bacteria;Bacteria;Bacteroidetes;Cytophagia;cytophagales;Cyclobacteriaceae | -0.324 | Axis 1 |
| Bacteria;Bacteria;Proteobacteria;Deltaproteobacteria;Desulfuronadales | 0.638 | Axis 2 |
| Bacteria;Bacteria;Chloroflexi;Anaerolineae;DRC31;Napoli-2B-44 BC07-2B-44 | 0.749 | Axis 2 |

**Table S7**. Significantly expressed gene functions in Elmer’s Beach metatranscriptomes

| **Function*** | **Relative Abundance June 3rd** | **Relative Abundance June 21st** |
| --- | --- | --- |
| 4-hydroxybenzoate 3-monooxygenase | 0.439 | 0.099 |
| 4,5-dihydroxyphthalate decarboxylase | 0.104 | 0.007 |
| acetoacetyl-CoA reductase | 12.341 | 12.030 |
| aldehyde dehydrogenase | 6.175 | 8.067 |
| benzaldehyde dehydrogenase | 0.578 | 0.130 |
| catechol 1,2-dioxygenase | 0.109 | 0.032 |
| cyclohexanone monooxygenase | 1.389 | 0.569 |
| cytochrome P450 | 0.364 | 0.165 |
| extradiol dioxygenase | 0.002 | 0.000 |
| flavin-binding family monooxygenase | 0.458 | 0.175 |
| gentisate 1,2-dioxygenase | 0.074 | 0.003 |
| homogentisate 1,2-dioxygenase | 1.274 | 0.915 |
| intradiol ring-cleavage dioxygenase | 0.002 | 0.000 |
| NAD+-dependent aryl-alcohol dehydrogenase | 0.726 | 0.341 |
| naphthalene 1,2-dioxygenase | 0.072 | 0.036 |
| oxaloacetate decarboxylase | 4.631 | 6.938 |
| PAH dioxygenase | 0.024 | 0.017 |
| protocatechuate 3,4-dioxygenase | 0.152 | 0.025 |

*****All functions are significant (p-value <0.02)

**Table S8:** Assembly of Metatranscriptomes from Elmer’s Beach

| Date | Raw | Assembled | Protein Annotated | 16S rRNA Raw | 16S rRNA Annotated |
| --- | --- | --- | --- | --- | --- |
| June 3rd | 368,657 | 346,455 | 202,425 | 460 | 129 |
| June 21st | 156,978 | 147,344 | 67,000 | 9,254 | 3,179 |
| June 29th | 2,901 | 2,750 | 666 | 676 | 180 |
