# Supplementary material for: Assessment of the Deepwater Horizon oil spill impact on Gulf coast microbial communities
Source: Front Microbiol. 2014 Apr 3;5:130. doi: 10.3389/fmicb.2014.00130 (PMC3982105; doi:10.3389/fmicb.2014.00130)
Supplement: Supplementary file 2 [file DataSheet2.PDF]

Supplemental Figures

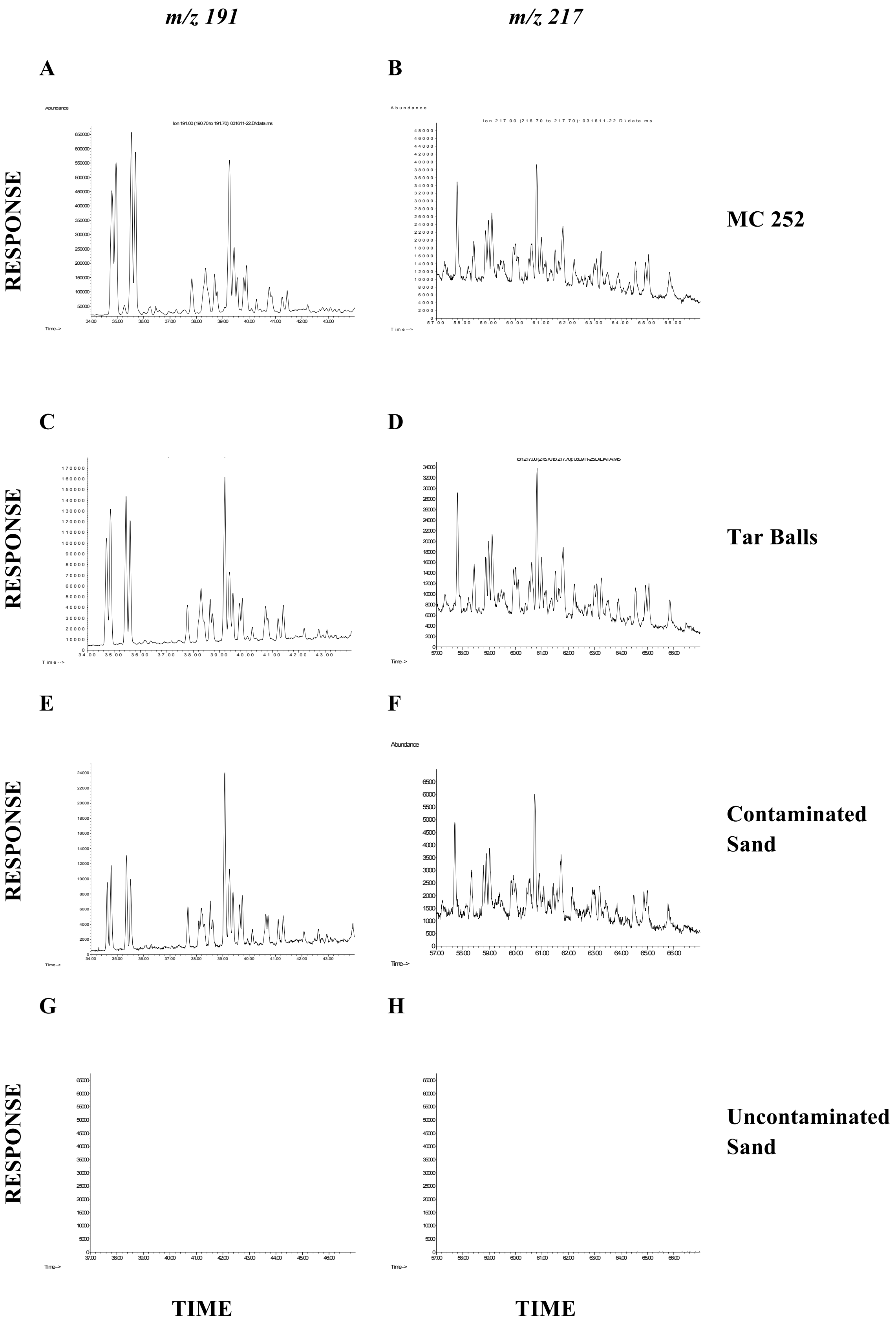

Figure S1. Typical results from SIM monitoring of m/z 191 Hopanes and m/z 217 steranes showing similarity in biomarker peaks for MC 252 source oil, tar balls, contaminated samples, and non-detect for uncontaminated sand. Panels **A, B** MC 252 oil; **C, D** Beached oil (sample EB03_Oil); **E, F** oil contaminated sand (EB14_3cm); **G, H** uncontaminated sand (EB02_3cm).

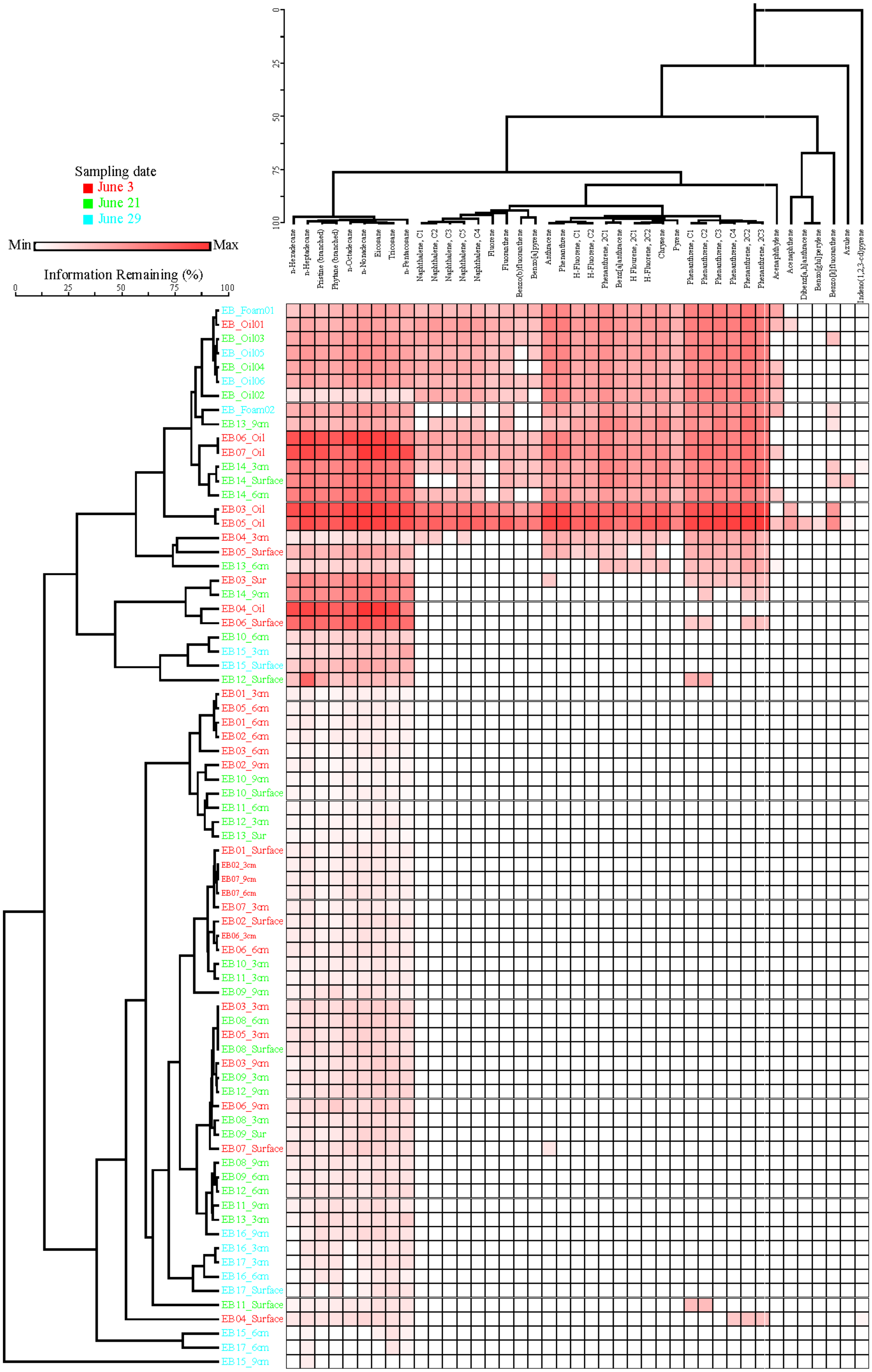

Figure S2. Clustering of sand and beached oil samples based on measured PAH and alkane compound composition. Chemical concentrations (µg/kg) were log(X+1) transformed and that matrix was used to create the two-way clustering dendrogram using the Bray-Curtis metric.

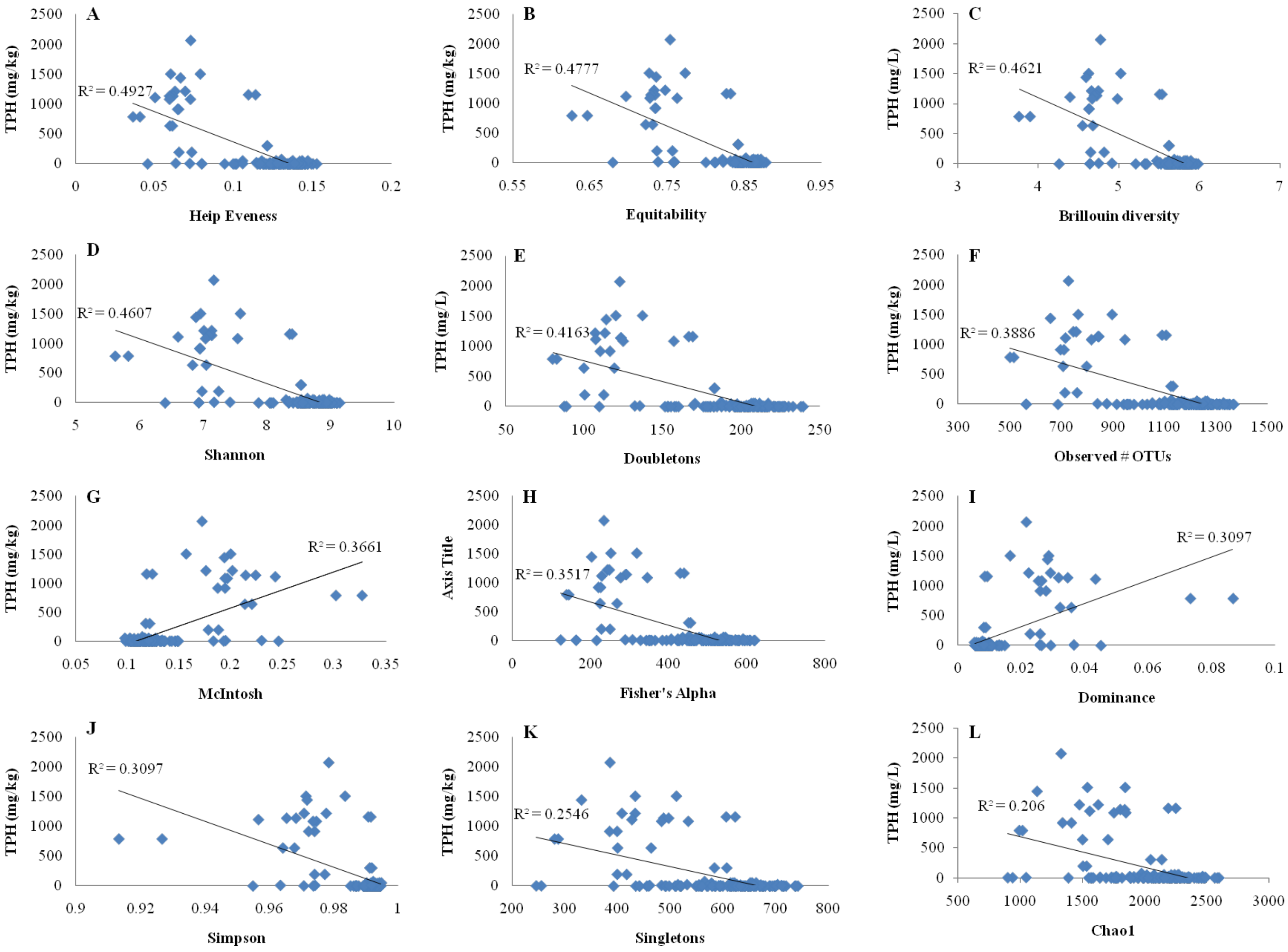

Figure S3. Linear correlations of alpha diversity metrics to TPH concentration in collected samples. (A) Heip evenness (B) Equitability (C) Brillouin diversity (D) Shannon index (E) Doubletons (F) Observed OTUs (G) McIntosh (H) Fisher's Alpha (I) Dominance (J) Simpson (K) Singletons (L) Chao1. All diversity calculations were performed within Qiime v1.0 software using rarified OTU tables generated using Pyrotagger. The minimum number of sequences used per sample was 5,000 sequences and 50 iterations were used.

A

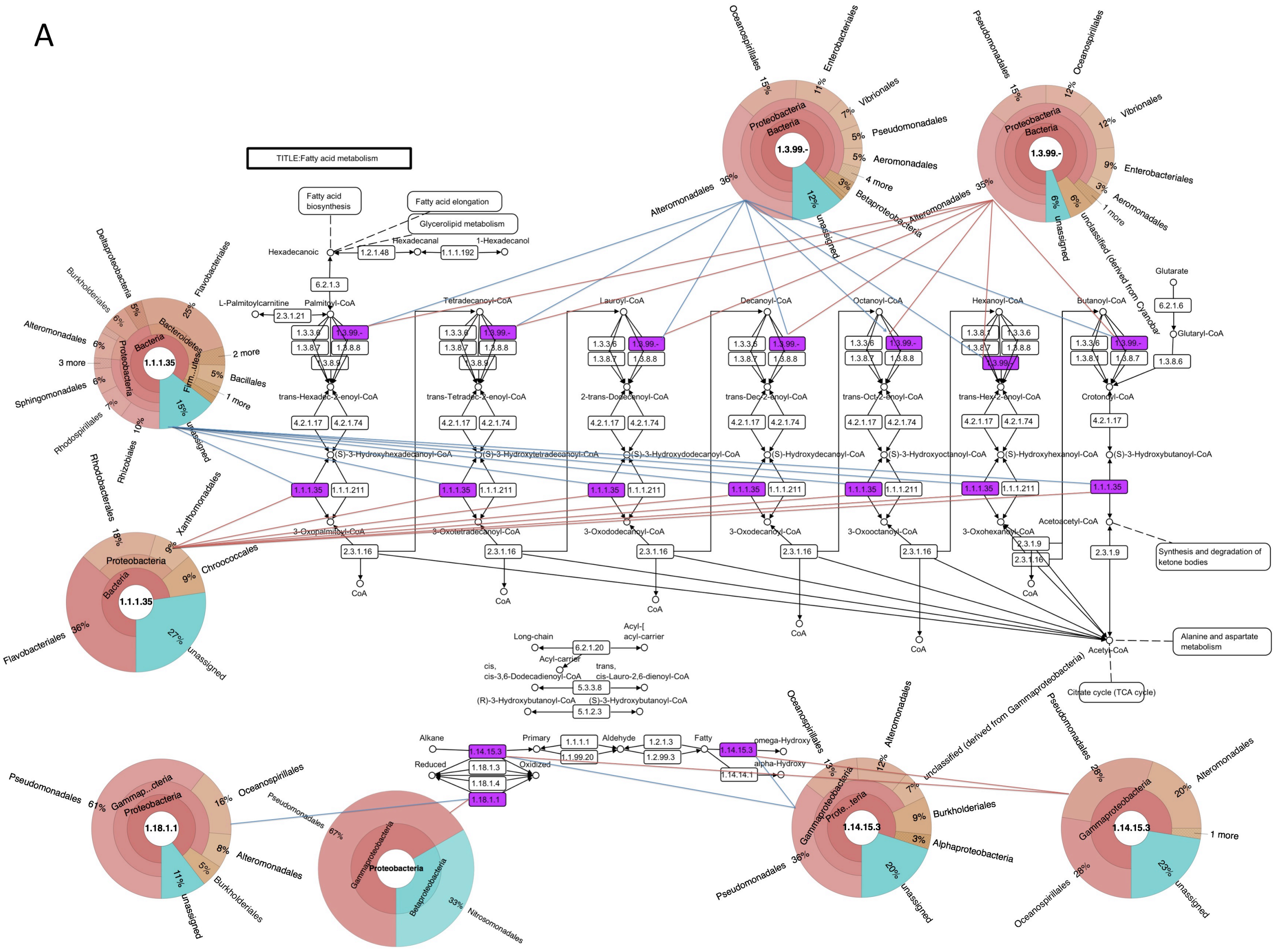

TITLE:Fatty acid metabolism
Fatty acid biosynthesis
Fatty acid elongation
Glycerolipid metabolism
Hexadecanoic
Hexadecanal
1-Hexadecanol
1.2.1.48
1.1.1.192
6.2.1.3
L-Palmitoylcarnitine
2.3.1.21
Palmitoyl-CoA
Tetradecanoyl-CoA
Lauroyl-CoA
Decanoyl-CoA
Octanoyl-CoA
Hexanoyl-CoA
Butanoyl-CoA
Glutarate
6.2.1.6
Glutaryl-CoA
1.3.8.6
1.3.3.6
1.3.8.7
1.3.8.8
1.3.8.9
1.3.99.-
trans-Hexadec-2-enoyl-CoA
trans-Tetradec-2-enoyl-CoA
2-trans-Dodecenoyl-CoA
trans-Dec-2-enoyl-CoA
trans-Oct-2-enoyl-CoA
trans-Hex-2-enoyl-CoA
Crotonoyl-CoA
4.2.1.17
4.2.1.74
(S)-3-Hydroxyhexadecanoyl-CoA
(S)-3-Hydroxytetradecanoyl-CoA
(S)-3-Hydroxydodecanoyl-CoA
(S)-Hydroxydecanoyl-CoA
(S)-3-Hydroxyoctanoyl-CoA
(S)-Hydroxyhexanoyl-CoA
(S)-3-Hydroxybutanoyl-CoA
1.1.1.35
1.1.1.211
3-Oxopalmitoyl-CoA
3-Oxotetradecanoyl-CoA
3-Oxododecanoyl-CoA
3-Oxodecanoyl-CoA
3-Oxooctanoyl-CoA
3-Oxohexanoyl-CoA
Acetoacetyl-CoA
2.3.1.16
2.3.1.9
CoA
Synthesis and degradation of ketone bodies
Acetyl-CoA
Alanine and aspartate metabolism
Citrate cycle (TCA cycle)
Long-chain
Acyl-[acyl-carrier
6.2.1.20
Acyl-carrier
cis, cis-3,6-Dodecadienoyl-CoA
trans, cis-Lauro-2,6-dienoyl-CoA
5.3.3.8
(R)-3-Hydroxybutanoyl-CoA
(S)-3-Hydroxybutanoyl-CoA
5.1.2.3
Alkane
1.14.15.3
Primary
1.1.1.1
1.1.99.20
Aldehyde
1.2.1.3
1.2.99.3
Fatty
omega-Hydroxy
alpha-Hydroxy
1.14.14.1
Reduced
Oxidized
1.18.1.3
1.18.1.4
1.18.1.1
1.3.99.-
Bacteria
Proteobacteria
Oceanospirillales 15%
Enterobacteriales 11%
Vibrionales 7%
Pseudomonadales 5%
Aeromonadales 5%
4 more
Betaproteobacteria 3%
unassigned 12%
Alteromonadales 36%
1.3.99.-
Bacteria
Proteobacteria
Pseudomonadales 15%
Oceanospirillales 12%
Vibrionales 12%
Enterobacteriales 9%
Aeromonadales 3%
1 more
unclassified (derived from Cyanobac
6%
unassigned 6%
Alteromonadales 35%
1.1.1.35
Bacteria
Proteobacteria
Bacteroidetes
Firm...utes
Flavobacteriales 25%
Deltaproteobacteria 5%
Burkholderiales 6%
Alteromonadales 6%
3 more
Sphingomonadales 6%
Rhodospirillales 7%
Rhizobiales 10%
unassigned 15%
2 more
Bacillales 5%
1 more
1.1.1.35
Bacteria
Proteobacteria
Rhodobacterales 18%
Xanthomonadales 9%
Chroococcales 9%
Flavobacteriales 36%
unassigned 27%
1.18.1.1
Proteobacteria
Gammap...cteria
Pseudomonadales 61%
Oceanospirillales 16%
Alteromonadales 8%
Burkholderiales 5%
unassigned 11%
Proteobacteria
Gammaproteobacteria
Betaproteobacteria
Pseudomonadales 67%
Nitrosomonadales 33%
1.14.15.3
Prote...teria
Gammaproteobacteria
Oceanospirillales 13%
Alteromonadales 12%
unclassified (derived from Gammaproteobacteria) 7%
Burkholderiales 9%
Alphaproteobacteria 3%
unassigned 20%
Pseudomonadales 36%
1.14.15.3
Gammaproteobacteria
Pseudomonadales 28%
Alteromonadales 20%
1 more
unassigned 23%
Oceanospirillales 28%

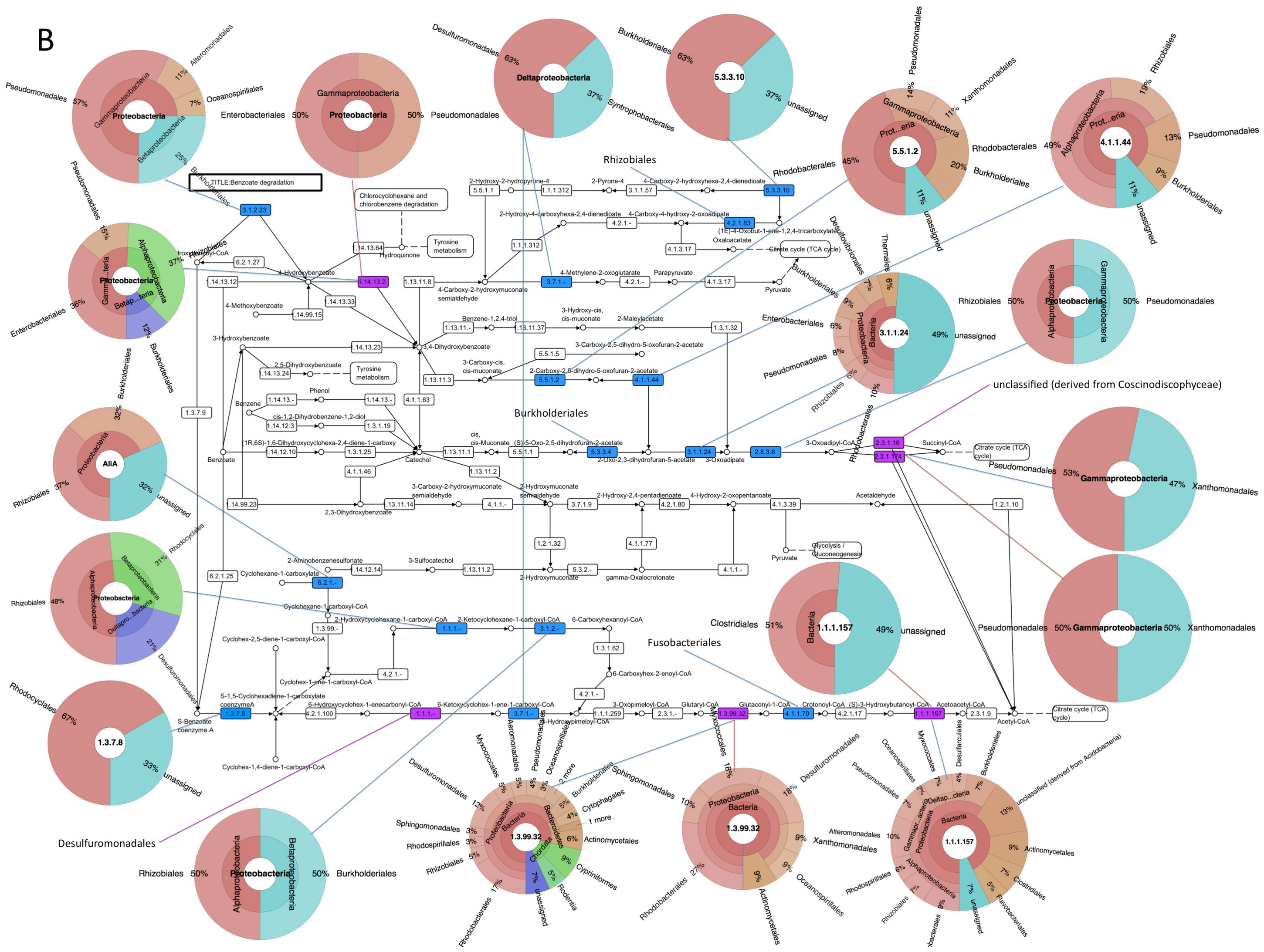

Figure S4. Fatty acid metabolism (**A**) and benzoate degradation (**B**) pathways. Assembled contigs are mapped to pathways from the KEGG database and colored in blue for June 3, red for June 21, and purple for presence in both time points. Pie charts indicating the best-hit taxonomic classification for each function were generated using Krona (See *Material and Methods*).

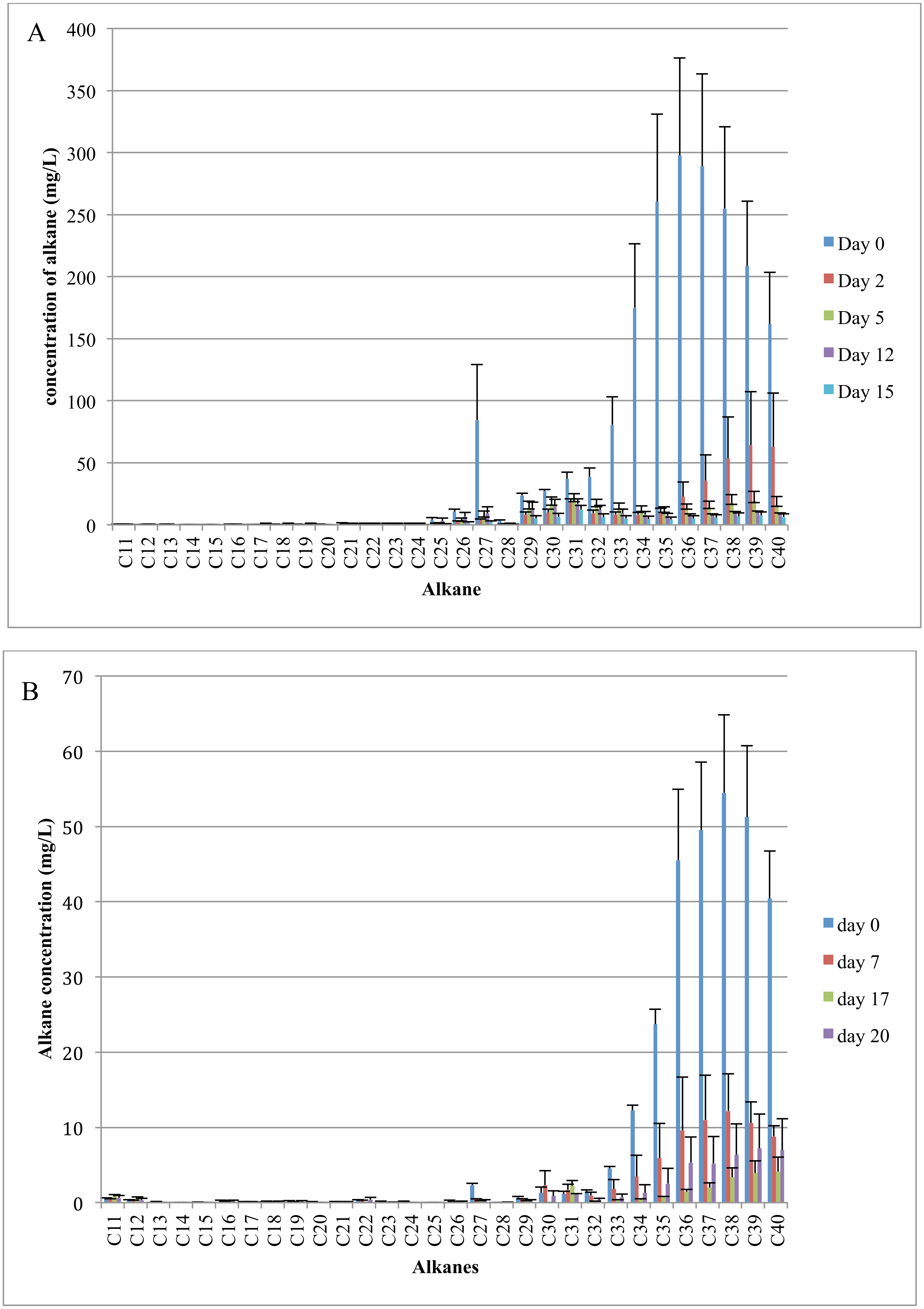

Figure S5. Degradation of alkane components by (A) Isolate 33- *Marinobacter spp*. (B) Isolate 36- *Roseobacter spp*.

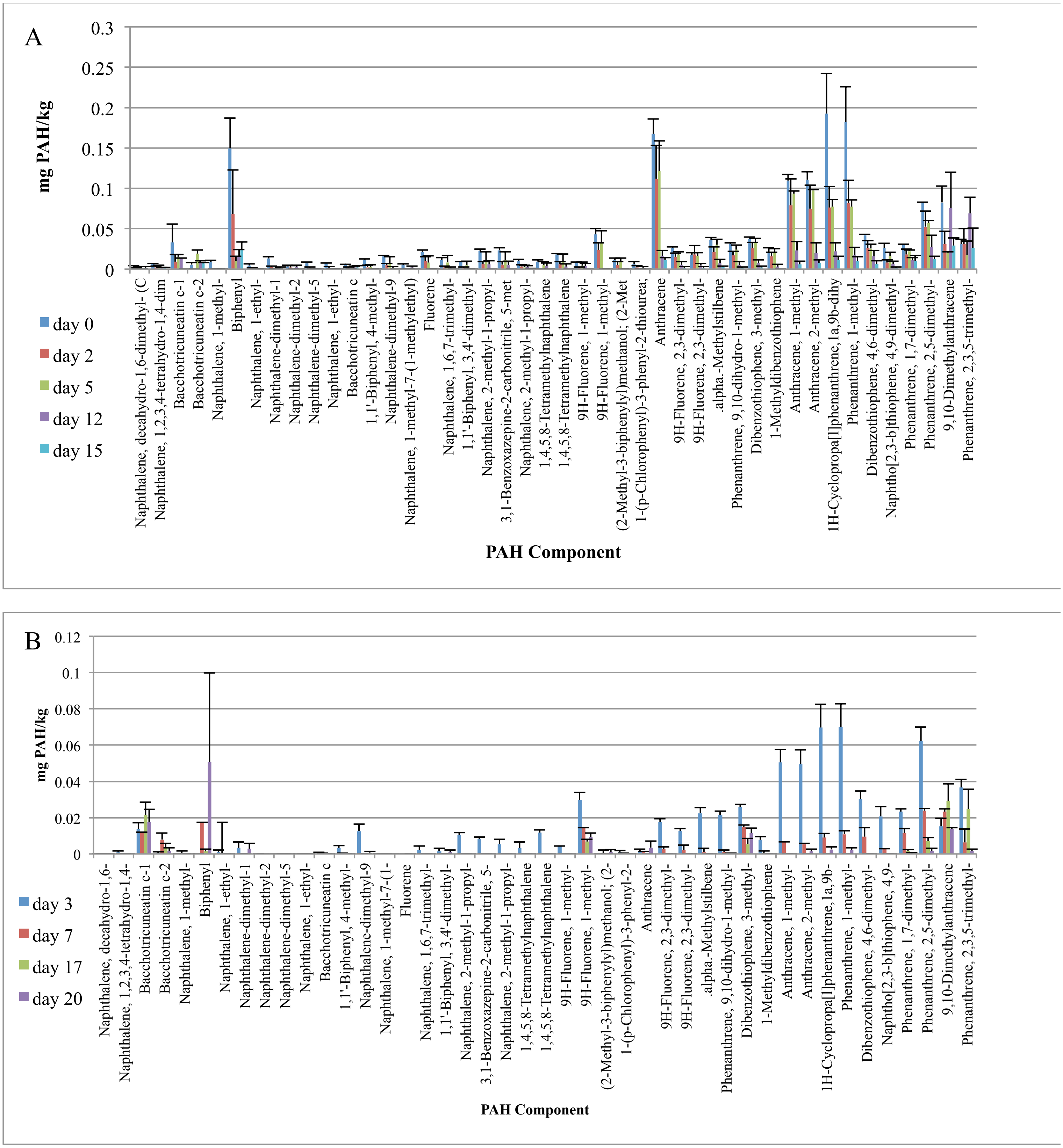

Figure S6. Degradation of PAH components by (A) Isolate 33- *Marinobacter spp*. (B) Isolate 36- *Roseobacter spp*.
